# Cardiac function in newborns of obese women and the effect of exercise during pregnancy. A randomized controlled trial

**DOI:** 10.1371/journal.pone.0197334

**Published:** 2018-06-01

**Authors:** Siri Ann Nyrnes, Kirsti Krohn Garnæs, Øyvind Salvesen, Anita Sharma Timilsina, Trine Moholdt, Charlotte Björk Ingul

**Affiliations:** 1 Department of Circulation and Medical Imaging, NTNU, Norwegian University of Science and Technology, Trondheim, Norway; 2 Department of Pediatrics, St. Olavs Hospital, Trondheim University Hospital, Trondheim, Norway; 3 Department of Public Health and General Practice, NTNU, Norwegian University of Science and Technology, Trondheim, Norway; 4 Department of Obstetrics and Gynecology, St. Olavs Hospital, Trondheim University Hospital, Trondheim, Norway; 5 Helse Midt-Norge RHF, Størdal, Norway; University of Missouri Columbia, UNITED STATES

## Abstract

**Background:**

The prevalence of maternal obesity is rising. Pre-pregnancy obesity is associated with later cardiovascular disease in the child and the underlying pathogenesis begins in early life. Therefore, pregnancy and infancy are key periods for potential intervention. The aim of this study was to study the cardiac function in newborns of obese women compared to newborns of normal-weight women, and to determine if exercise intervention during pregnancy could have an effect on cardiac function of newborns to obese women.

**Material and methods:**

Fifty-five pregnant women, 51 obese (BMI ≥ 30 kg/m^2^) and four overweight (BMI 28–30 kg/m^2^), were randomized to an exercise training group (n = 27) or a control group (standard maternity care, n = 28). From gestational week 14 until delivery participants in the intervention group were offered supervised training sessions three times weekly. In addition, they were told to exercise at home once weekly. All newborns had an echocardiogram performed 1–3 days and 6–8 weeks after delivery. The results were compared with newborns of normal weight women (n = 20, standard maternity care).

**Results:**

Newborns of obese women had an impaired systolic and diastolic cardiac function with reduced global strain, strain rate, tissue Doppler velocities and a thicker intraventricular septum at birth and after 6–8 weeks after delivery compared to newborns of normal weight women. Exercise had no statistically significant effect on either of the cardiac function parameters. The mean (± standard deviation) adherence to the exercise protocol was 1.3 ± 0.8 sessions per week for supervised training and 0.8 ± 0.7 sessions per week for home-based exercise training.

**Conclusions:**

Newborns of obese women had reduced cardiac function and thicker intraventricular septum compared to newborns of normal weight women. Exercise training during pregnancy had no significant effect, potentially due to a low number of subjects and low adherence to the exercise protocol.

**Trial registration:**

ClinicalTrials.gov NCT01243554.

## Introduction

### Background and objectives

The prevalence of maternal obesity is rising [1], and if trends continue, global obesity prevalence will surpass 21% in women by 2025 [2]. The mean body mass index (BMI) in women has now reached 32.2 kg/m^2^ in areas of the world where obesity is most prevalent, but vary substantially by region [2]. Maternal obesity creates an unfavorable in utero environment regarding genetic, hormonal and biochemical factors, influencing fetal growth and development [3].

Recent reports emphasize that fetal life plays an important role regarding future susceptibility to disease, including cardiovascular disease [4, 5]. Pre-pregnancy obesity is associated with later cardiovascular health for the child [6, 7]. Moreover, maternal obesity during pregnancy is linked to premature mortality from cardiovascular events when the child reaches adult life [8]. In addition, obese women are at significantly increased risk of bearing children with a range of congenital heart defects [9, 10]. The mechanisms associating maternal obesity with cardiovascular disease in the child are multifactorial and complex [6]. Subclinical cardiac changes measured by echocardiographic techniques such as speckle tracking and tissue Doppler have been reported already in fetal life of obese mothers [11, 12], and in the newborn of obese women with pre-gestational type 2 diabetes [13].

Pregnancy and infancy are key periods for potential lifestyle intervention to prevent future cardiovascular disease. Exercise is considered safe and beneficial for most women with healthy pregnancies, as well as for their fetus [14–16]. Exercise during pregnancy could be potentially even more beneficial for obese women [17], and it is recommended that they gradually build up to 20–30 minutes of moderate intensity training per day [18]. There is emerging interest in how exercise can influence the fetus and child, and maybe even prevent future health problems [19]. However, there is still only limited evidence of the effect of exercise during pregnancy on cardiac function in the newborns. The aim of this study was to investigate the cardiac function in newborns of obese women (NOW) compared to newborns of normal weight women (NNW), and to determine the effect of an exercise intervention during pregnancy on cardiac function of NOW.

## Materials and methods

### Design

This study called the NeoETIP (Neonates in the Exercise Training in Pregnancy) trial, was a sub study of the Exercise Training in Pregnancy (ETIP) trial, which was a single center, parallel group randomized controlled trial [20]. The trial was carried out at The Norwegian University of Science and Technology, NTNU, and St. Olavs hospital, University hospital in Trondheim, Norway, with participant recruitment from September 2010 to March 2015 [21, 22].

This trial was approved by the Regional Committee for Medical and Health Research Ethics, REC Central (Reference 2010/1522), and the ETIP trial is registered in ClinicalTrials.gov (NCT01243554). The NeoETIP study was approved through a change request to the ethical committee (approval date April 2011), and the change was registered in ClinicalTrials.gov. The pregnant women gave informed consent for their newborns being examined. Additional changes made to the ETIP study protocol affecting the NeoETIP study, was that the inclusion criteria pre-pregnancy BMI ≥ 30 kg/m^2^ was changed to BMI ≥ 28 kg/m^2^ (March 2013). This change was done to accommodate slow recruitment in the trial.

### Participants

Obese and a few overweight women were recruited through the ongoing randomized ETIP trial [20, 21]. In addition, we recruited a group with normal weight mothers for comparison in the NeoETIP sub study. Inclusion criteria were pre-pregnancy body mass index (BMI) ≥ 28 kg/m^2^ for the NOW groups and BMI between 18.5 and 25 kg/ m^2^ for normal weight women (NNW group). Common for all groups were age ≥ 18 years, carrying a singleton viable fetus at 11–14 gestational weeks. Exclusion criteria in the NOW groups were diseases affecting participation, high risk for preterm delivery, and regular exercise training (twice or more weekly) in the period before inclusion.

Women in the ETIP study were recruited in early pregnancy through primary healthcare center, local advertisement, Google advertisements and by notices enclosed with invitations for routine ultrasound scans at St. Olavs hospital. The normal weight women were recruited in early pregnancy through primary healthcare centers and local advertisement.

At the time of recruitment and before randomization and participation, the women received written information and signed an informed consent.

### Interventions

Women in the exercise group were offered supervised exercise sessions at St. Olavs hospital three times weekly. The supervised sessions consisted of 35 minutes of moderate intensity endurance exercise (~ 80% of maximal capacity, corresponding to Borg scale 12–15) and 25 minutes of strength training (resistance training for large muscle groups and the pelvic floor muscles), from pregnancy week 14 until delivery. In addition, the women were told to follow a 50-minute home exercise program once weekly (35 minutes of endurance training and 15 minutes of strength training). Details regarding the exercise intervention in this study are published previously [21]. The exercise sessions were in accordance to guidelines from the American College of Obstetrics and Gynecologists [23]. The normal weight pregnant women and the obese pregnant women in the control group received standard maternity care.

### Outcomes

#### Cardiac function and size

Cardiac function was measured using echocardiography. The echocardiographic exams were scheduled to 1–3 days after delivery, and at 6–8 weeks of age. The study participants that did not turn up for the scheduled examination received a reminder with a new appointment. A full echocardiogram was performed by an experienced pediatric cardiologist (SAN, first author) with a Vivid 7 scanner (GE Vingmed Ultrasound, Horten, Norway) using a GE 7s phased-array transducer (GE healthcare, USA). Morphology and ventricular function were assessed. Three cine loops from three standard left ventricular (LV) apical planes (four-chamber, two-chamber and long-axis), right ventricle (RV) and LV parasternal view were recorded in B- mode and tissue Doppler mode. Conventional Doppler flow parameters were measured as well as tissue Doppler velocities. The images were processed and analyzed using Echo Pac. B-mode mean frame rate was 111.4/s (range 72/s and 188/s).

LV diameter, septum thickness and fractional shortening (FS) were measured from two-dimensional targeted M-mode echocardiographic tracings in the parasternal long axis, according to the criteria of the American Society of Echocardiography and the European Association of Cardiovascular Imaging [24].

Mitral annular-plane systolic excursion (MAPSE) and tricuspid annular-plane systolic excursion (TAPSE) were assessed by M-mode from an apical four-chamber view by placing the cursor at a right angle to the atrioventricular junction, and the maximum amplitude of displacement was measured [25, 26].

Systolic (S’), early diastolic (E’) and late diastolic (A’) peak pulse wave tissue Doppler velocities were measured from the LV annuli of the apical four-chamber (mitral and lateral) and two-chamber (inferior and anterior) view, and for the right ventricle (RV) from the tricuspid lateral annuli [27].

Longitudinal two-dimensional (2D) strain and strain rate by speckle-tracking echocardiography (STE) were performed, and digital loops were acquired from the three apical views. The best-quality 2D image cardiac cycle was selected. A semi-automatic method (EchoPAC, GE, Milwaukee, Wisconsin) was used offline to define the region of interest and the internal border of the myocardium was outlined. Segments that failed to track were manually adjusted, and segments that subsequently failed to track were excluded. Region of interest was adjusted manually if necessary to fit the average of the myocardial thickness. The software uses ECG to define end-diastole. 2D strain and strain rate were automatically calculated by the software algorithm at each frame throughout the heart cycle as a curve in six different segments for each apical view. LV global longitudinal strain/strain rate were defined as the average of peak longitudinal strains from a 16 LV segments model [28]. RV global longitudinal strain/strain rate were calculated from the RV free wall.

#### Blood pressure and heart rate in the newborns

Blood pressure and ECG data in the children was collected by a cardiac nurse or a pediatric cardiologist (SAN). These examinations were done just before the echocardiographic examination. The blood pressure was ideally measured in the resting state of the child, and if this was achieved and the measurement was considered of good quality, only one measurement was performed [29]. Up to three measurements were performed in participants where resting state was more challenging, and then the lowest measurement was chosen. We performed the blood pressure measurements using a CASMED 740 Monitor, a multi parameter monitor (ScanMed as Norway). The non-invasive blood pressure was measured using the oscillometric technique determining the systolic, diastolic and mean arterial pressure, and pulse rate. The systolic and diastolic blood pressures were registered for analysis. The appropriate sized cuff was chosen, i.e. the smallest cuff size covering least 2/3 of the right upper arm was selected. The EKG was performed using a Philips PageWriter Touch EKG Machine (Philips Medical Systems, USA)

### Sample size

Sample size calculations by the independent samples T-test were based on the expected difference between the neonates of the obese mothers (NOW) and the neonates of the normal weight mothers (NNW). With an estimated group difference (NOW versus NNW) of 1mm (4 *vs* 3 mm) for neonatal interventricular septum thickness, standard deviation (SD) of 1, α = 0.05 and power of 0.8, 16 women in each group were needed [30]. To allow for a dropout proportion of 20%, 20 participants in in each group were required. As we were not able to find evidence for an expected difference between the NOW exercise group (NOWe) and NOW control group (NOWc) regarding cardiac function, this comparison was considered more as a hypothesis generating and as a base for future sample size calculations.

### Randomization and allocation

Pregnant women with a pre-pregnancy BMI ≥ 28 kg/m^2^ were randomized to an exercise training group or a control group (standard maternity care). Trial participants were randomized 1:1 to exercise or control using a computer random number generator developed and administrated at the Unit for Applied Clinical Research at NTNU to generate the random allocation.

Newborns of obese women (n = 55) were examined by echocardiography at 1–3 days of age and at 6–8 weeks of age. The second timepoint was chosen to be sure the transition phase was completed and that pulmonary resistance was fallen to the normal level. The results were compared with corresponding echocardiograms from newborns of normal weight women (n = 20).

### Blinding and echocardiographic analysis

The ECG and blood pressure measurements in the neonates were collected by a cardiac nurse or a pediatric cardiologist (SAN), both blinded for exercise group allocation. The same pediatric cardiologist (SAN) performed the echocardiographic exams. The pediatric cardiologist was not blinded to general clinical information regarding the neonates, and was informed about the women belonging to the NNW group.

A research assistant trained to analyze echocardiographic data (AST) remeasured and plotted all the echocardiographic data from Echopac for further statistical analysis. She had no information regarding the neonates, the maternal group, or the exercise group allocation. These data and analyses were used in the statistical comparison between groups. The statistician was also blinded for group allocation and all clinical information regarding the neonates. The cardiologist who contributed to the reproducibility measurements (C.B.I, last author) of LV global longitudinal strain was blinded regarding clinical information and group allocation.

### Statistical methods

Basic clinical data was tested by independent samples t-tests and expressed as mean ± SD.

The time-development of different cardiac function variables for the three groups (NOWc, NOWe and NNW) was assessed with mixed linear models. To account for repeated measurements, participant ID was included as a random effect. The independent correlation structure was chosen for the error terms. In comparison between groups, we report mean values with 95% confidence intervals (CI). We considered p-values < 0.05 as significant. The analyses were performed using R version 2.13.1 and IBM SPSS Statistics 23.

## Results

### Participant flow and numbers analyzed

**Fig 1:**
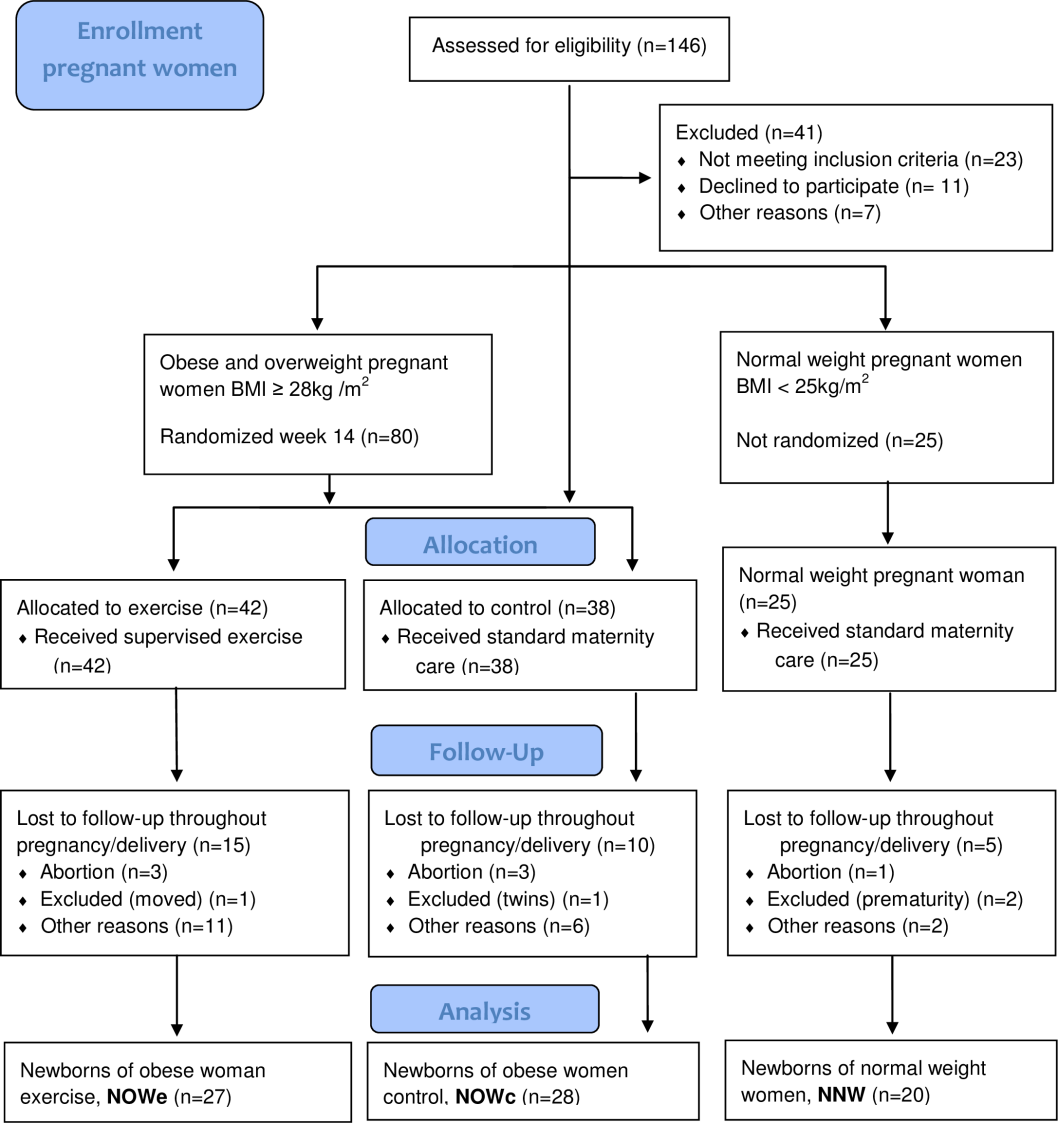
Flowchart of the pregnant women in the NeoETIP trial (CONSORT flow diagram). Women who were lost to follow-up throughout the pregnancy- and delivery period, categorized in the “other reasons”- group, either forgot or chose not to give notice of birth to the study group regarding the scheduled cardiac examination of the newborn. Neonates in the Exercise Training in Pregnancy (NeoETIP), Body mass index (BMI), Number (N).

**Fig 2:**
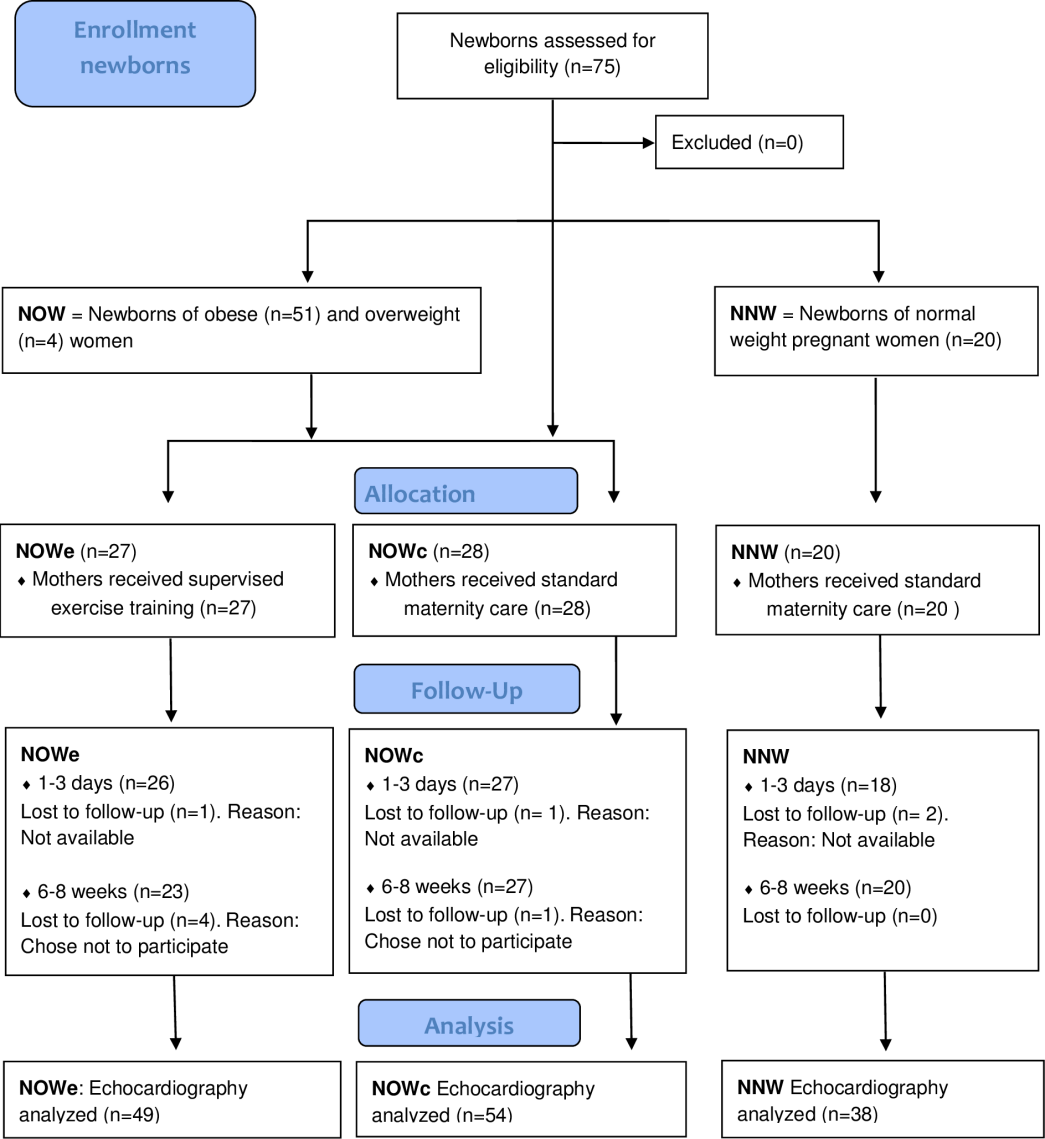
Flowchart of the newborns in the NeoETIP trial (CONSORT flow diagram). The final study population: Newborns of normal weight women (NNW), Newborns of obese women (NOW), NOWe randomized to exercise and NOWc randomized to control (standard maternity care). The newborns underwent cardiac examination 1–3 days of age and 6–8 weeks of age. Neonates in the Exercise Training in Pregnancy (NeoETIP), Body mass index (BMI), Number (N).

Details regarding the enrollment and participant flow of the pregnant women are presented in Fig 1. In total, 75 newborns were included in the NeoETIP study; 27 NOWe, 28 NOWc and 20 NNW. A total of 141 cardiac exams including echocardiography were performed. Four newborns in the NOW group were not available for the first scheduled cardiac examination: One was born at another hospital, another was born when the echocardiographer was not available, and two forgot to give the study group notice of birth. Two newborns in the NNW group were not available for the first examination, one was born at another hospital and one mother did not give the study group notice of birth. For the second timepoint exam (6–8 weeks), five participants (NOW group only) who completed cardiac exam at birth chose not to participate (Fig 2).

The first cardiac assessment (age in days) was performed: Median (first quartile, third quartile) 3.0 (2.0, 3.0) for the NNW group, 2.0 (1.0, 3.0) for the NOWe group and 2.0 (1.0, 3.0) for the NOWc group. The second cardiac assessment (age in weeks) was performed: Median (first quartile, third quartile) 8.4 (7.1, 12.1) for the NNW group, 8.6 (7.8, 9.6) for the NOWe group and 7.7 (7.1, 9.0) for the NOWc group. The study participants who did not turn up for the scheduled control received a reminder with a new appointment: The median dates of control were in the upper part of the control interval, with all outliers after scheduled control (not normally distributed).

### Recruitment

The study examinations of neonates of normal weight woman were performed between June 2012 and November 2013. The scheduled study examinations of neonates of obese women for the NeoETIP sub study was performed between May 2011 and February 2014. Inclusions of newborns stopped before inclusions in the ETIP-study were completed, because we had examined the number of participants intended for the NeoETIP sub study. Neonates with congenital heart defects (both NOW and NNW groups) received standard clinical follow-up after the study period. The latest follow up echocardiographic examination in the study group was performed in April 2017.

### Baseline data—The pregnant women

Four women in the NOW group were classified as overweight at baseline, two of them in the exercise group (BMI 29.7 and 29.1), and two in the control group (BMI 28 and 29.5) The remaining women in the NOW group had a pre-pregnancy BMI ≥ 30, and were classified as obese. Pre-pregnancy BMI was 33.4 ± 3.4 in the NOW exercise group, 34.9 ± 3.9 in the NOW control group, and 21.0 ± 2.3 in the normal weight group (NNW). There was no statistically significant difference between the NOW exercise and control group regarding BMI (p = 0.14). The NOW exercise group had an age of 31.1 ± 3.0 years, the NOW control group had an age of 31.3 ± 4.6 and the normal weight group had an age of 31.2 ± 4.1 years. The baseline characteristics regarding the pregnant women are presented in Table 1.

**Table 1:** Baseline characteristics–The pregnant women.

Details regarding the women	Normal weight women (n = 20)	Obese women (exercise, n = 27)	Obese women (control, n = 28)
Pre pregnancy BMI (kg)	21.0 ± 2.3	33.4 ± 3.4	34.9 ± 3.9
Body weight at full term (kg)	74.5 ± 11.4	104.9 ± 11.2	109.4 ± 15.8
Mean age (years)	31.2 ± 4.1	31.1 ± 3.0	31.3 ± 4.6
Gestational diabetes (week 34–37)*	0 (0.0%)	4 (15.0%)	7 (25.0%)
Smoking	0 (0.0%)	4 (15.0%)	4 (14.3%)
First pregnancy	11 (55.0%)	15 (55.6%)	15 (53.6%)
Second pregnancy	6 (30.0%)	9 (33.3%)	10 (35.7%)
Third pregnancy	3 (15.0%)	3 (11.1%)	2 (7.1%)
Forth pregnancy	0 (0.0%)	0 (0.0%)	1 (3.6%)
Mode of delivery: Operational	2 (10.0%)	4 (14.8%)	5 (17.9%)
Mode of delivery: Caesarean	2 (10.0%)	8 (28.6%)	4 (14.3%)
*Mode of delivery: Normal*	16 (80.0%)	15 (55.6%)	19 (67.8%)

Data are presented as mean ± standard deviation or number of participants (percent). Body mass index (BMI), Blood pressure (BP), Kilograms (kg)

* International Association of Diabetes and Pregnancy Study Groups (IADPSG) and the World Health Organization 2013 (WHO 2013) definition: Fasting plasma glucose ≥ 5.1 mmol/l or 120-min plasma glucose ≥ 8.5 mmol/l.

### Basic clinical data—The newborns

Mean gestational age at birth were 40.3 ± 1.1 weeks in the NNW group, 39.4 ±1.7 weeks in the NOWc group and 39.2 ± 1.3 weeks in the NOWe group. Mean newborn birth weight was 3.6 ± 0.5 kg in the NNW group, 3.9 ± 0.4 kg in the NOW control group and 3.7 ± 0.6 kg in the NOW exercise group. The corresponding birth lengths were 49.8 ± 1.9 cm (NNW), 51.1 ± 2.0 cm (NOWc) and 50.5 ± 1.5 cm (NOWe). At the second cardiac examination (between 6 and 8 weeks of age) the mean weight/length was: 5.8 ± 1.1 kg /61.6 ± 4.4 cm (NNW), 5.7 ± 1.0 kg/59.8 ± 2.7 cm (NOWc) and 5.5 ± 0.5 kg/60.1 ± 4.5 cm (NOWe). Blood pressure and heart rate (measured by ECG) are presented in Table 2.

**Table 2:** Blood pressure and heart rate.

Variables	Age 1–3 days		Age 6–8 weeks			
Groups	NNW Mean (95% CI)	NOW Exercise Mean (95% CI)	NOW Control Mean (95% CI)	NNW Mean (95% CI)	NOW Exercise Mean (95% CI)	NOW Control Mean (95% CI)
ECG (beats/min)	118 (110 to 126)	123 (116 to 129)	122 (115 to 128)	142 (134 to 150)	143 (136 to 151)	148 (141 to 154)
Systolic BP (mmHg)	80 (74 to 86)	80 (74 to 85)	82 (77 to 87)	83 (77 to 89)	83(78 to 89)	88 (83 to 93)
**Diastolic BP (mmHg)**	46 (41 to 51)	46 (41 to 50)	47 (43 to 51)	54 (49 to 58)	51 (46 to 55)	52 (40 to 56)

Data are presented as mean and 95% confidence interval (CI). Newborns of normal weight women (NNW), Newborns of obese women (NOW), Heart rate measured by printed 12-lead Electrocardiogram (ECG in beats/min), Blood pressure (BP)

Five newborns needed admission to the neonatal intensive care unit after birth; Two newborns in the NOW exercise group, three in the NOW control group and no one in the NNW group. In the exercise group, one was admitted due to prematurity (week 34) and one due to meconium aspiration. In the NOW control group one newborn was admitted due to hypoglycemia and infection, one due to asphyxia caused by shoulder dystocia, complicated by a humerus fracture, and one due to persistent pulmonary hypertension.

### Outcomes and estimations

#### Cardiac function and size

Newborns of obese women had impaired systolic and diastolic cardiac function with reduced global longitudinal strain in the left and right ventricle, strain rate, tissue Doppler velocities and MAPSE. They also had thicker interventricular septum at birth, and still at 6–8 weeks after delivery compared to newborns of normal weight mothers (Tables 3 and 4).

**Table 3:** Cardiac function and size—Echocardiographic measurements.

Cardiac variables	Age 1–3 days			Age 6–8 weeks		
Groups	NNW Mean (95% CI)	NOW exercise Mean (95% CI)	NOW control Mean (95% CI)	NNW Mean (95% CI)	NOW exercise Mean (95% CI)	NOW control Mean (95% CI)
GLS LV (%)	-23.2 (-24.7 to -21.6)	-17.2 (-18.5 to 15.9)	- 16.9 (-18.1 to -15.6)	- 24.2 (-25.6 to -22.7)	-21.0 (-22.4 to -19.7)	-20.1 (-21.4 to -18.9)
GLSR LV (-1)	-2.0 (-2.1 to -1.9)	-1.6 (-1.7 to -1.5)	- 1.7 (-1.7 to -1.6)	-2.2 (-2.4 to -2.1)	-1.8 (-1.9 to -1.7)	-1.8 (-1.9–1.7)
GLS RV (%)	-26.9 (-29.3 to- 24.4)	-20.9 (-23.0 to -18.9)	-18.6 (-20.6 to -16.7)	-31.6 (-33.9 to -29.3)	-22.74 (-24.9 to -20.5)	-21.30 (-23.3 to -19.3)
GLSSR RV (-1)	-2.7 (-3.0 to -2.4)	-1.90 (-2.1 to -1.7)	-1.7 (-2.0 to -1.5)	-3.2 (-3.5 to -2.9)	-2.5 (-2.8 to -2.2)	-2.1 (-2.4–1.9)
S’ LV (cm/s)	6.4 (5.9 to 6.9)	4.4 (3.9 to 4.8)	4.2 (3.7 to 4.6)	8.2 (7.7–8.7)	6.1 (5.6–6.6)	6.2 (5.7 to 6.6)
E’ LV (cm/s)	7.4 (6.6 to 8.3)	5.8 (5.1 to 6.5)	5.5 (4.7 to 6.2)	12.2 (11.3 to 13.1)	9.6 (8.8 to 10.4)	8.8 (8.1 to 9.6)
A’ LV (cm/s)	7.0 (6.2 to 7.7)	6.1 (5.4 to 6.8)	6.4 (5.7 to 7.0)	9.6 (8.8 to 10.4)	8.6 (7.9 to 9.4)	8.5 (7.8 to 9.2)
TAPSE (mm)	10.1 (9.3 to 10.8)	9.3 (8.6 to 9.9)	9.0 (8.3 to 9.6)	15.2 (14.4 to 15.9)	13.9 (13.2 to 14.6)	14.2 (13.6 to14.9)
MAPSE (mm)	5.0 (4.7 to 5.3)	3.9 (3.6 to 4.2)	3.9 (3.6 to 4.2)	7.7 (7.4 to 8.1)	6.5 (6.2 to 6.8)	6.3 (6.0 to 6.6)
Septum (mm)	4.4 (3.8 to 5.1)	5.0 (4.5 to 5.5)	5.6 (5.1 to 6.1)	4.7 (4.1 to 5.3)	6.0 (5.4 to 6.5)	5.9 (5.4 to 6.4)
LVIDd (cm)	1.9 (1.8 to 2.0)	1.8 (1.8 to 1.9)	1.8 (1.7 to 1.9)	2.3 (2.2 to 2.4)	2.2 (2.1 to 2.3)	2.2 (2.1 to 2.3)
**FS (%)**	**38.1 (35.8 to 40.6)**	**37.0 (35.0 to 39.0)**	**36.2 (34.2 to 38.1)**	**37.8 (35.5 to 40.0)**	**35.2 (33.1 to 37.3)**	**35.0 (33.0 to 37.0)**

Data are presented as mean and 95% confidence interval (CI). Newborns of normal weight women (NNW), Newborns of obese women (NOW), Left ventricle (LV), Right ventricle (RV), S’ peak systolic tissue Doppler velocity (TDV), E’ peak early diastolic TDV, A’ peak late diastolic TDV, mitral annular-plane systolic excursion (MAPSE), tricuspid annular-plane systolic excursion (TAPSE), intraventricular septum in diastole (IVSd), left ventricular internal diameter in enddiastole (LVIDd), fractional shortening (FS).

**Table 4:** Between-group comparisons: Cardiac function and size.

Cardiac variables	Age 1–3 days			Age 6–8 weeks		
Group comparisons	NOWe—NOWcMD (95% CI)	NNW—NOWcMD (95% CI)	NNW—OWeMD (95% CI)	NOWe—OWcMD (95% CI)	NNW—NOWcMD (95% CI)	NNW—NOWeMD (95% CI)
GLS LV (%)	-0.3 (-2.1 to 1.5)	-6.3 (-8.2 to -4.3)	-6.0 (-8.0 to -4.0)	-0.9 (-2.7 to 0.9)	-4.1 (-6.0 to -2.2)	-3.2 (-5.2 to -1.2)
GLSR LV (-1)	0.1 (-0.1 to 0.2)	-0.3 (-0.5 to -0.1)	-0.4 (-0.6 to -0.2)	0.0 (-0.2 to 0.2)	-0.4 (-0.6 to -0.3)	-0.4 (-0.6 to -0.2)
GLS RV (%)	-2.3 (-5.1 to 0.6)	-8.2 (-11.4 to -5.1)	-5.9 (-9.1 to -2.8)	-1.4 (-4.4 to 1.5)	-10.3 (-13.3 to -7.2)	-8.9 (-12.1 to -5.7)
GLSSR RV (-1)	-0.2 (-0.5 to 0.2)	-1.0 (-1.4 to -0.6)	-0.8 (-1.2 to -0.4)	-0.4 (-0.7 to 0.0)	-1.1 (-1.5 to 0.7)	-0.7 (-1.1 to -0.3)
S’ LV (cm/s)	0.2 (-0.4 to 0.8)	2.3 (1.6 to 2.9)	2.1 (1.4 to 2.7)	-0.1 (-0.7 to 0.6)	2.0 (1.4 to 2.7)	2.1 (1.4 to 2.8)
E’ LV (cm/s)	0.3 (-0.7 to 1.4)	2.0 (0.8 to 3.1)	1.6 (0.5 to 2.8)	0.8 (-0.3 to 1.8)	3.3 (2.2 to 4.5)	2.6 (1.4 to 3.8)
A’ LV (cm/s)	-0.3 (-1.2 to 0.7)	0.6 (-0.4 to 1.6)	0.8 (-0.2 to 1.9)	0.1 (-0.9 to 1.1)	1.1 (0.0 to 2.1)	0.9 (-0.2 to 2.0)
TAPSE (mm)	0.3 (-0.61 to 1.2)	1.1 (0.1 to 2.1)	0.8 (-0.2 to 1.8)	-0.4 (-1.3 to 0.6)	1.0 (0.0 to 1.9)	1.3 (0.3 to 2.3)
MAPSE (mm)	0.0 (-0.4 to 0.4)	1.1 (0.7 to 1.5)	1.1 (0.7 to 1.5)	0.2 (-0.2 to 0.6)	1.4 (1.0 to 1.9)	1.2 (0.8 to 1.6)
Septum (mm)	-0.6 (-1.4 to 0.10)	-1.2 (-2.0 to -0.4)	-0.6 (-1.4 to 0.3)	0.1 (-0.7 to 0.9)	-1.2 (-2.0 to—0.4)	-1.3 (-2.1 to -0.5)
LVIDd (cm)	0.0 (-0.1 to 0.1)	0.1 (-0.0 to 0.2)	0.1 (-0.0 to 0.2)	0.0 (-0.1 to 0.1)	0.1 (-0.0 to 0.2)	0.1 (-0.0 to 0.2)
**FS (%)**	0.8 (-2.0 to 3.6)	2.0 (-1.1 to 5.1)	1.2 (-1.9 to 4.3)	0.2 (-2.8 to 3.1)	2.7 (-0.3 to 5.8)	2.6 (-0.5 to 5.7)

Data are presented as mean and 95% confidence interval (CI). Newborns of normal weight women (NNW), Newborns of obese women exercise (NOWe), Newborns of obese women control (NOWc) Left ventricle (LV), Right ventricle (RV), S’ peak systolic tissue Doppler velocity (TDV), e’ peak early diastolic TDV, A’ peak late diastolic TDV, mitral annular-plane systolic excursion (MAPSE), tricuspid annular-plane systolic excursion (TAPSE), intraventricular septum in diastole (IVSd), left ventricular internal diameter in enddiastole (LVIDd), fractional shortening (FS).

**Fig 3:**
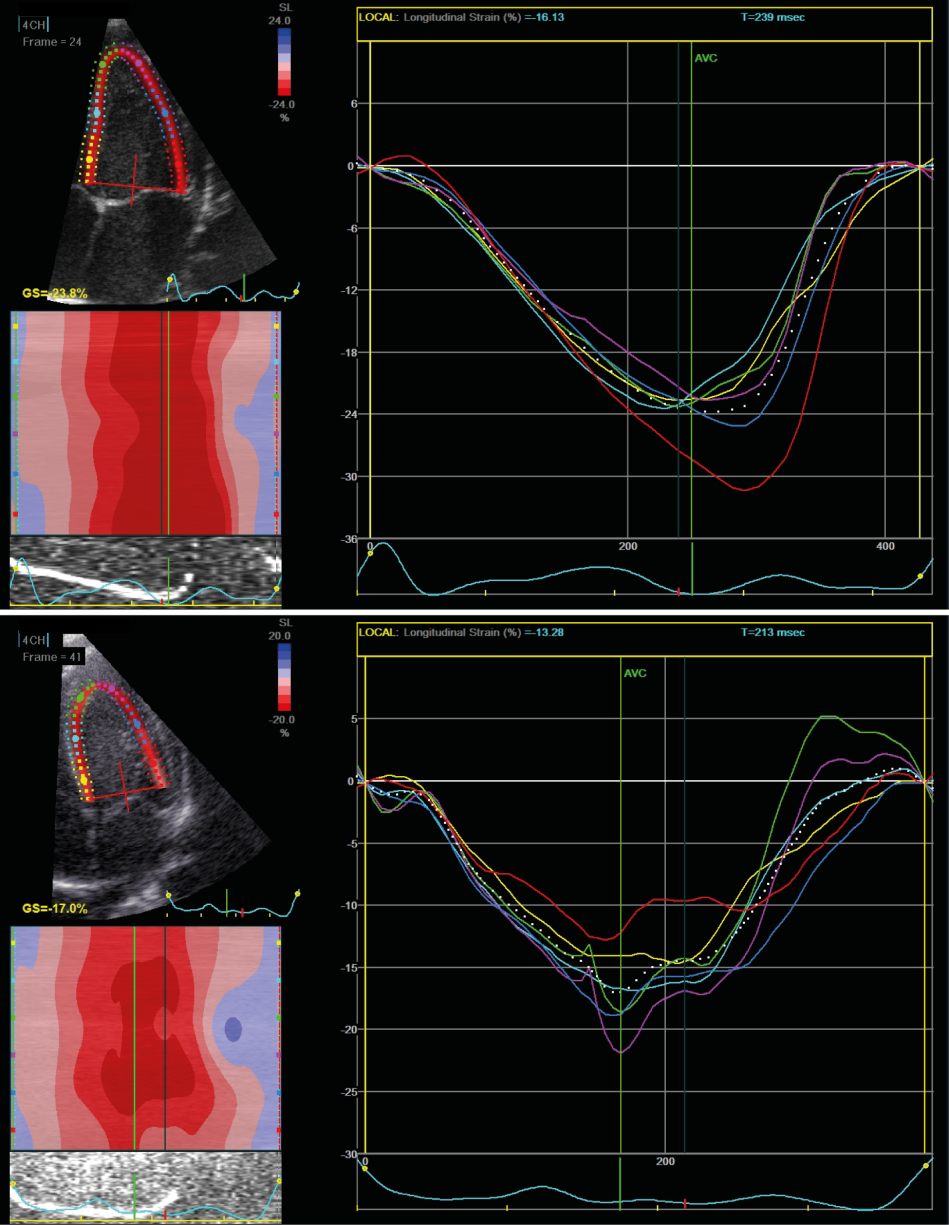
Cardiac function–Longitudinal strain in the left ventricle. Examples of apical 4 chamber views with speckle tracking in a newborn of a normal weight women (NNW, the upper image) and a newborn of an obese women in the control group (NOWc, lower image) at 6–8 weeks.

**Fig 4:**
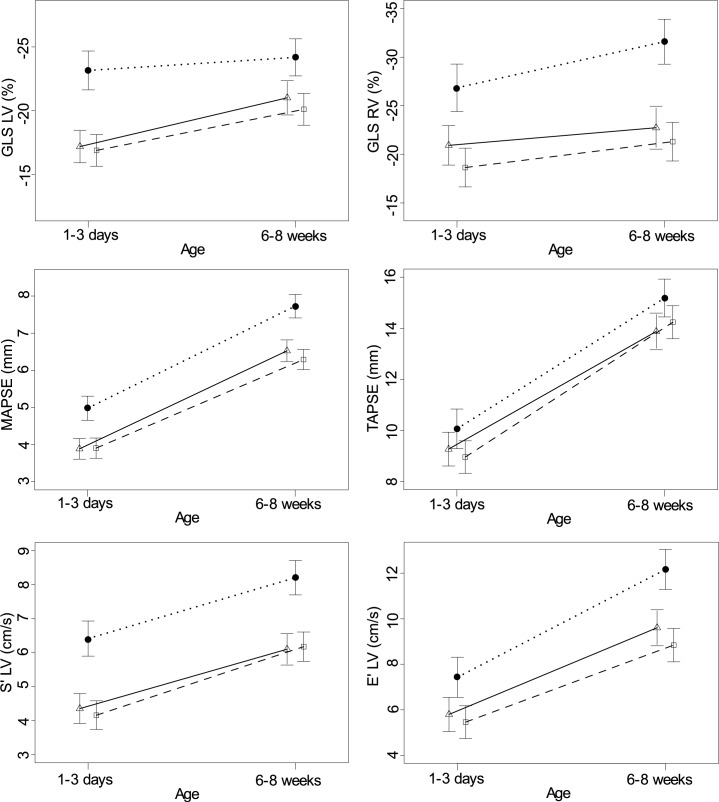
Cardiac function–A comparison between groups over time. The figure demonstrates the difference between the groups with 95% confidence intervals and the physiologic increase in cardiac function over time during the first weeks of life. The symbols represent the following groups: The filled circle (●) represents newborns of normal weight women (NNW), and a dotted line is drawn between the circles. The triangle (Δ) represents newborns of obese women in the exercise group (NOWe), and a solid line is drawn between the triangles. The square (□) represents newborns of obese women in the control group (NOWc), and a broken line is drawn between the squares. Left ventricle (LV), Right ventricle (RV), S’ peak systolic tissue Doppler velocity (TDV), E’ peak early diastolic TDV, mitral annular-plane systolic excursion (MAPSE), tricuspid annular-plane systolic excursion (TAPSE).

Fig 3 shows an example of LV global longitudinal strain (GLS) curve in a newborn in the NNW and one in the NOWc group. Fig 4 provides a graphic comparison between groups over time for GLS in the left and right ventricle, S’, E’, MAPSE and TAPSE. This figure demonstrates that the differences between groups persist to the second cardiac examination at 6–8 weeks of age. In addition, the figure illustrates the physiological increase in cardiac function over time during the first weeks of life.

Exercise had no statistically significant effect on cardiac function. However, there was a small but consistent tendency towards slightly better cardiovascular measures in the exercise group compared to the control group (Fig 4, Tables 3 and 4). The number of exercise sessions per week in the NOWe group was 1.3 ± 0.8 for supervised sessions, and 0.8 ± 0.7 for home-based sessions. In the exercise group, 15 of 27 women managed to exercise per protocol.

#### Reproducibility

We assessed the reproducibility of measurement of LV global longitudinal strain (GLS) from a random sample of 20 echocardiographic examinations (10 NOW and 10 NNW) at 1–3 days after birth (n = 10) and 6–8 weeks after birth (n = 10). Data were reanalyzed by the same investigator (C.B.I.), and by a second investigator (A.S.T.), giving complete sets for intra- and interobserver variability. The intraclass correlation coefficient was estimated at 0.96 for both intra- and inter observer measurements.

#### Ancillary analyses—Congenital heart defects

Only minor cardiac defects were detected. In the newborns of the obese women, four newborns had a small secundum atrial septal defect (ASD), all underwent spontaneous closure before three years of age. Three newborns had a small muscular ventricular septal defect (VSD) which closed spontaneously before two years of age. One patient had persistent pulmonary hypertension of the newborn, which required admission to the intensive care unit, however, the pulmonary pressure at the second cardiac examination in the study (6–8 weeks) was fully normalized.

In the NNW group, none had hemodynamic significant congenital heart defects. However, one newborn had a secundum ASD, (small residual defect at three years of age) and one newborn had a small muscular VSD which closed spontaneously before control at two years of age.

## Discussion

### Main findings

The major findings in this study were that newborns of obese women had reduced cardiac function and increased interventricular septal thickness compared to newborns of normal weight women. The alterations in cardiac function and size were subclinical in all patients. However, our findings may be of clinical significance, since recent reports demonstrates that pre-pregnancy obesity is associated with later cardiovascular health for the child [8–10].

### Cardiac function

Our findings of reduced cardiac function in neonates of obese women are consistent with previous reports [12, 13, 31], but adds extended observation period and showed sustained impaired cardiac function several weeks after delivery. We recently published data from the same cohort on fetal cardiac function, where reduced cardiac function was observed already at 14 weeks’ gestation [11]. In the present study, we demonstrated that the reduced cardiac function in the NOW group persisted at birth and 6–8 weeks after birth. We also found that the physiological increase in cardiac function over time was similar among newborns of obese and normal weight women (Fig 4).

In comparison with published data from Al-Biltagi et al. [32] in newborn of women with pre-gestational and gestational diabetes, the values on cardiac function from the NOW group in the present NeoETIP study were better. Al-Biltagi et al [32] reported values for GLS in the groups with pre-gestational diabetes (-10.4%) and gestational diabetes (-13.1%), which are low compared to what we found in the NOWe (-17.2%) and NOWc (-16.9%) groups. Their corresponding normal values were also slightly lower (-19.1%) than ours (-23.2%). However, this study did not specify the maternal BMI. Likewise, data on maternal BMI were not provided in the published normal values of left ventricular function in term neonates during the transitional period [27]. This study reported values for GLS of -21.7 ± 1.9 (day 1) and -21.2 ± 1.8 (day 2). Our values from the NNW group corresponds to their upper normal values [27].

Our normal group may be considered as “supernormal”, as it only consisted of normal weight mothers. The mentioned studies [27, 32], utilized the same ultrasound equipment, and they examined the neonates at the same time point as our first assessment, thus the data should otherwise be comparable.

The cardiopulmonary transitional phase from fetal to neonatal life may influence the measures of cardiac function. However, Jain et al.[27] found that most functional measures, when evaluated after 12 hours of age, remained unchanged over the next two days. Still, the combination of the closure of fetal shunts, the changes in cardiac output as well as systemic and pulmonary preload, afterload and resistance must be kept in mind, since many of the measured echocardiographic parameters seem to be load-dependent [33]. But load should affect both newborns of obese and normal weight mothers equally.

Animal studies have generated knowledge regarding mechanisms of developmental fetal programming leading to reduced fetal cardiac function due to maternal obesity [34–36]. Maternal obesity is associated with hormonal, biochemical and genetic alterations which can influence fetal programming of cardiovascular disease [3, 6]. The hyperglycemia, insulin resistance, diabetes, hypertriglyceridemia, elevated inflammatory markers, increased oxidative stress and endothelial dysfunction associated with maternal obesity are potentially harmful for the fetal heart [37].

### Cardiac size and structure

Maternal obesity has been shown to increase the risk of congenital heart defects [9, 10, 38], as is also the case for maternal diabetes [39, 40]. In our study, we only detected minor cardiac defects, 8/55 (15%) in the NOW group and 2/20 (10%) in the NNW group. It is known that maternal diabetes leads to increased cardiac intraventricular septal thickness in the child [41]. The hypertrophy is reported to be a transient disorder, which regresses spontaneously during the first six months of life with normalization of insulin [42]. Our findings, with significantly increased intraventricular septal thickness, and to a lesser extent thickening of the left ventricular free wall in the NOW group, are like the findings reported in children of diabetic mothers [32].

### Exercise

Exercise during pregnancy had no statistically significant effect on cardiac function of the neonates in the NeoETIP study. The main reason is probably low adherence to exercise, and partly due to too few study subjects. However, there was a small, but consistent tendency towards improved cardiac function in the exercise group. The ETIP study found a reduced incidence of gestational diabetes mellitus and lower systolic blood pressure in late pregnancy among women randomized to the exercise training program [21], but no influence on basic maternal or neonatal outcomes at delivery [22]. The effects of exercise are thought to be positive for the fetal cardiac development during pregnancy [14], however no absolute consensus exists [15].

### Limitations, strengths and further perspectives

The main limitations in this study were the low number of participants in each group and suboptimal adherence to supervised exercise. However, the exercise intervention lead to a reduction in gestational diabetes mellitus and systolic blood pressure in late pregnancy [21], as well as reduced circulating insulin levels tree moths postpartum[43]. This shows that even a small amount of exercise has positive effects. Since we observed a tendency towards better neonatal cardiac function in the exercise group, we speculate if improved exercise adherence, prolonged intervention period and a higher number of participants would have revealed a statistically significant difference. Moreover, addition of exercise both before pregnancy and in the first trimester would be the ideal to affect fetal programming by creating a healthy environment in utero during the critical time for organ development [44]. Future studies should address these issues. An additional limitation of the study is the fact that the normal weight pregnant women were not randomized.

The number of subjects with minor congenital heart defects among the newborns of the obese mothers in our study were high, compared to what would be expected in a normal population. However, these defects had no hemodynamic significance, and probably had minimal influence of the measurements of cardiac function. In the NNW group, two outliers met later for the second examination than the outliers of the NOW groups. This could explain the fact that NNW had slightly higher mean weight than NOW at the second examination. However, patient size (length and weight) at the second examination were not significantly different between groups, and we believe that these outliers do not significantly influence our measurements on cardiac function.

One of the strengths in our data was the longitudinal follow up with up to five repeated measures of cardiac function and size from fetal life (gestational week 14–20 and 32) [11], to newborn at birth and 6–8 weeks after birth. In accordance with novel data on future risk of cardiovascular disease in the children of obese mothers [8], it would be of interest with an additional cardiac exam, to determine if the differences persist into childhood/adolescence.

### Generalizability and clinical relevance

This study added new information about cardiac function in neonates of obese mothers compared to newborns of normal weight mothers. Our data indicate that neonatal normal values for cardiac function and size mirror the obesity prevalence in the maternal population studied. It is therefore expected to be significant variations in normal values by regions reflecting the prevalence of obesity. Based on our findings, neonatal normal data for echocardiographic function measures should ideally not include values from obese mothers, or at least report the mean BMI of the mothers. This is novel knowledge, together with the finding of sustained impaired cardiac function after an extended observation period.

The prevalence of maternal obesity has rapidly increased [1, 2], and there is a need to establish strategies to prevent the associated risk for the mother and child. New knowledge that might increase the motivation to a healthier lifestyle, could be the key to make obese women adhere to an exercise program. Information regarding the effect of obesity on the neonatal heart could strengthen this motivation. Ideally the lifestyle change should start before pregnancy to gain the optimal protective effect to alter the fetal programming. In our study population, the exercise was well tolerated by the women, fetus and newborn, and this is consistent with other studies [45]. The health benefit from lifestyle intervention should be communicated clearly in the counseling of pregnant woman and women who plan a pregnancy.

Increased research efforts to understand the relationship between maternal obesity, the effects of exercise, and fetal cardiovascular development are needed, as well as knowledge of how to motivate a sedate population for lifestyle changes during pregnancy. To identify the mechanisms for early cardiovascular disease, and to determine whether the adverse effects of maternal obesity can be modified by lifestyle changes, are highly warranted. Even though our study did not provide certain evidence regarding the exercise intervention, it should encourage and guide future research.

## Conclusions

Newborns of obese women had reduced cardiac function and thicker interventricular septum compared to newborns of normal weight women. Exercise training during pregnancy had no statistic significant effect, maybe due to few study subjects and low adherence to the exercise protocol. Future studies should focus on pre-pregnancy exercise as well.

## Supporting information

S1 Protocol ETIP trial (Exercise Training in Pregnancy)–the original ETIP protocol, ethics committee approved. (DOCX)

S2 Protocol ETIP trial–The published (TRIALS) protocol. (PDF)

S3 Protocol NeoETIP (Neonates in the Exercise Training in Pregnancy)–Original NeoETIP protocol, ethics committee approved. (PDF)

S1 Text Consort checklist. **The NeoETIP trial** (Neonates in the Exercise Training in Pregnancy).
(DOC)

S2 Text The Regional Committee for Medical and Health Research Ethics (REC central) approval. The ETIP **(**Exercise Training in Pregnancy) trial.
(PDF)

S3 Text The Regional Committee for Medical and Health Research Ethics (REC central) approval. Approval of the change request regarding the NeoETIP study.
(PDF)

S4 Text Information given to obese and overweight pregnant women. (PDF)

S5 Text Information given to normal weight pregnant women. (PDF)

S1 Appendix SPSS file with raw data from the NeoETIP study. (SAV)
