# Supplementary material for: Cardiac function in newborns of obese women and the effect of exercise during pregnancy. A randomized controlled trial
Source: PLoS One. 2018 Jun 1;13(6):e0197334. doi: 10.1371/journal.pone.0197334 (PMC5983429; doi:10.1371/journal.pone.0197334)
Supplement: S2 Protocol — (PDF) [file pone.0197334.s002.pdf]

Moholdt et al. Trials 2011, 12:154
http://www.trialsjournal.com/content/12/1/154

TRIALS

STUDY PROTOCOL Open Access

Exercise Training in Pregnancy for obese women (ETIP): study protocol for a randomised controlled trial

Trine T Moholdt[1,3*], Kjell Salvesen[2], Charlotte B Ingul[3], Torstein Vik[4], Emily Oken[5] and Siv Mørkved[1]

Background: Both maternal pre-pregnancy obesity and excessive gestational weight gain are increasing in prevalence and associated with a number of adverse pregnancy outcomes for both mother and child. Observational studies regarding physical activity in pregnancy have found reduced weight gain in active mothers, as well as reduced risk of adverse pregnancy outcomes. There is however a lack of high quality, randomized controlled trials on the effects of regular exercise training in pregnancy, especially those with a pre-pregnancy body mass index (BMI) at or above 30 kg/m^2.

Methods: We are conducting a randomised, controlled trial in Norway with two parallel arms; one intervention group and one control group. We will enroll 150 previously sedentary, pregnant women with a pre-pregnancy BMI at or above 30 kg/m^2. The intervention group will meet for organized exercise training three times per week, starting in gestation week 14 (range 12-16). The control group will get standard antenatal care. The main outcome measure will be weight gain from baseline to delivery. Among the secondary outcome measures are changes in exercise capacity, endothelial function, physical activity level, body composition, serum markers of cardiovascular risk, incontinence, lumbopelvic pain and cardiac function from baseline to gestation week 37 (range 36-38). Offspring outcome measures include anthropometric variables at birth, Apgar score, as well as serum markers of inflammation and metabolism in cord blood.

Discussion: The results of this trial will provide knowledge about effects of regular exercise training in previously sedentary, obese pregnant women. If the program proves effective in reducing gestational weight gain and adverse pregnancy outcomes, such programs should be considered as part of routine pregnancy care for obese women.

Trial Registration: ClinicalTrials.gov: NCT01243554

Background

In the Unites States over one-third of reproductive age women are obese (body mass index (BMI) ≥ 30 kg/m^2) and another 29% are overweight (BMI 25.0-29.9 kg/m^2) [1]. Maternal obesity is associated with a number of adverse outcomes during and after pregnancy, such as gestational diabetes, preeclampsia, caesarean delivery and children born large for gestational age [2], as well as increased risk for childhood obesity among offspring. In addition, over 60% of overweight women gain more than recommended during pregnancy [3]. As gestational weight gain is directly associated with maternal weight retained during the postpartum period [3] as well as with offspring adiposity in childhood [4] and in early adulthood [5], excess gestational weight gain could accelerate the obesity epidemic. Current recommendations say that pregnant women should exercise with moderate intensity for 30 minutes or more on most, if not all, days of the week [6]. In general, women are not active enough during pregnancy and women who have a high pre-pregnancy BMI are even less likely to be physically active [7].

Today's knowledge about the importance of exercising regularly in controlling weight gain in pregnancy is

* Correspondence: trine.moholdt@ntnu.no
[1]Department of Public Health and General Practice, Norwegian University of Science and Technology, Trondheim, Norway
Full list of author information is available at the end of the article

BioMed Central
© 2011 Moholdt et al; licensee BioMed Central Ltd. This is an Open Access article distributed under the terms of the Creative Commons Attribution License (http://creativecommons.org/licenses/by/2.0), which permits unrestricted use, distribution, and reproduction in any medium, provided the original work is properly cited.

mainly based on results from observational studies [4,8-11]. Previous randomized controlled trials of physical activity during pregnancy are few in number and have had varying results. For example, Clapp et al [12] found significantly less weight gain in women who were randomized to high volume of exercise in late pregnancy, compared to moderate or low volumes, whereas others have found no significant effects of exercise training on gestational weight gain [13-15]. The reasons for the divergent results in these randomized trials could be low compliance, high drop out rates and inadequate number of participants in some of the studies, as well as differences in the mode, frequency, intensity and duration of the exercise training. A reduced risk of excessive weight gain has been found in women randomized to lifestyle counselling programs combining diet and exercise [16,17]. In such programs however, it is hard to say if it is the exercise component or other factors that make the women gain less weight in the intervention groups. Thus, there is a lack of good quality randomized controlled trials, assessing short and long term effects of physical activity in pregnancy on mothers and offspring. Especially, there is a need for studies looking at potential effects of regular exercise training during pregnancy in obese mothers.

In this paper, we describe the methods of the Exercise Training in Pregnancy (ETIP) trial, a randomized, controlled trial of physical activity during pregnancy.

Methods

Objectives

The primary objective of the ETIP trial is to test the hypothesis that obese pregnant women who exercise in addition to usual pregnancy care will have a lower gestational weight gain compared to women who receive usual care only.

Secondary, the ETIP trial will investigate the effects of exercise training on various pregnancy-related complications, such as insulin resistance, lumbopelvic pain, urinary and fecal incontinence, pelvic floor muscle dysfunction, and prolonged labour. We will also assess possible effects of exercise training on cardiopulmonary parameters as cardiac function, submaximal oxygen uptake, lactate threshold, heart rate recovery, endothelial function, and blood pressure, measured as changes in these parameters from early to late pregnancy. Offspring variables include Apgar score, weight, length and head circumference at delivery, prevalence of large for gestational age (LGA) and small for gestational age (SGA), blood pressure, and body composition, and cord blood markers of inflammation and insulin resistance.

Our hypothesis is that regular exercise training in pregnancy will reduce gestational weight gain and pregnancy-related complications. We also hypothesise that exercise will increase physical capacity, heart rate recovery and endothelial function, and reduce blood pressure, compared to the control group. Regarding offspring variables, we hypothesise that exercise training will reduce the prevalence of LGA, blood pressure, cord blood markers of inflammation and insulin resistance, as well as improve body composition, and Apgar score.

Participants and setting

We invite pregnant women with self-reported pre-pregnancy BMI ≥ 30 kg/m^2 to participate in the trial. Women are eligible if they are 18 years or older, with a singleton live fetus at an 11-14 weeks ultrasound scan. Exclusion criteria are pregnancy complications, high risk for preterm labour or diseases that could interfere with participation, and habitual exercise training (twice or more weekly). The women are given information about the project and will be recruited through the ordinary visits at general practitioners and midwives, and at outpatient clinics at the hospitals. Also, information about the study and invitation to participate is sent along with the invitation to come for routine ultra sound scan in week 18.

The trial will be conducted at the Norwegian University of Science and Technology and the St.Olavs Hospital, Trondheim University Hospital. The recruitment started in September 2010 and will continue until the needed number of participants is included, anticipated until the end of 2012. Participating women get infant food worth $85 US dollars.

Randomisation and allocation

After initial assessments, the women will be randomly assigned to either intervention or control (1:1 randomisation, Figure 1). Allocation is performed by a web-based randomisation system developed and administered by another unit at the university to ensure blinding. The randomisation will be in blocks with varying block size.

Intervention

We invite the training group to participate in an exercise program that we have specially designed for pregnancy, including aerobic activity, specific exercises for stabilization of the lower back and pelvis, and strength exercises for the pelvic floor muscles that we have specially designed for pregnancy. We conduct training groups for a total of 60 minutes four times weekly at the hospital. We ask the women to come to exercise training a minimum of twice weekly between 14 and 37 weeks of gestation. We also encourage the women to come for exercise training even after week 37. The endurance training consists of a 10 minute warm-up followed by walking on treadmills for 25 minutes. The intensity will be moderate, reaching ~80% of their

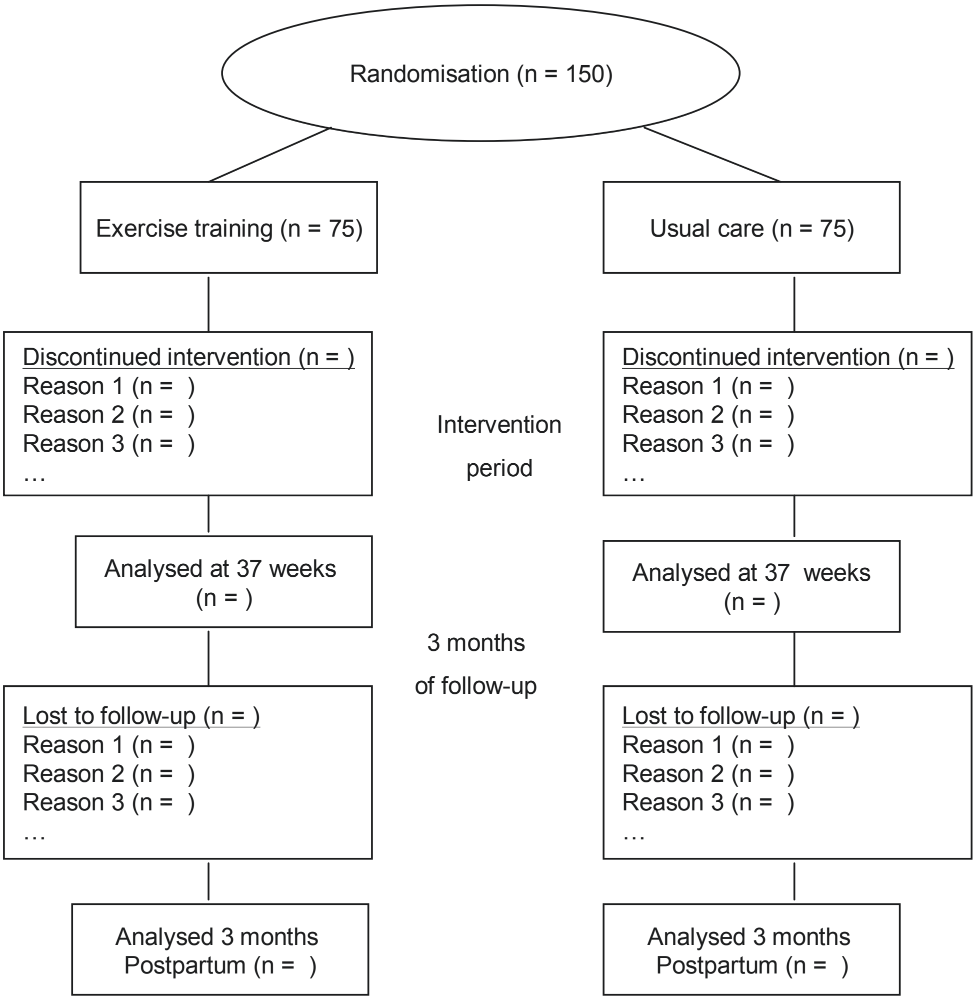

Figure 1 Flow-chart.

maximal capacity in periods (corresponding to Borg scale12-15) [18].

In addition, we instruct intervention women in a 50 minute home exercise program which we encourage them to complete at least once per week (35 minutes endurance training and 15 minutes strength exercises). We also teach pelvic floor muscle exercises to do daily. We also recommend the women to be physically active in every-day life. Adherence is strongly emphasized and registered in the women's personal training diary and the reports from the persons leading the training groups. During the training period, the subjects will go through motivational interviewing [19]. This is a client-centred therapeutic method to enhance readiness for change and to elicit the client's own motivations for change. Each woman will go through a 30 minute session of motivational interviewing in each trimester. They will also receive a weight gain curve that shows the recommended weight gain throughout pregnancy, based on 2009 Institute of Medicine guidelines [20]. The training protocol follows recommendations from the Norwegian Health Directory [21] and the American College of Obstetrics and Gynecology [6]. Specific adjustments are made to the exercise program if needed (for example by using a stationary bike instead of treadmill walking and by modifying the strength exercises to the actual strength level of the participants).

Women in the control group will receive the customary regular consultations with midwife, general practitioner or obstetrician. They are not discouraged from exercising on their own. Neither group will receive special recommendations about diet, beyond what is given through standard antenatal care. In Norway, the pregnancy care is free of charge. Routine prenatal visits are done by general practitioners, midwives, or a combination of the two, and are usually undertaken in gestational weeks 8-12, 24, 28, 32, 36, 38, 40, and 41. In addition, women are invited to an ultrasound scan is

gestational week 18. There is currently no knowledge about the actual advices about physical activity that pre-natal care providers give to pregnant women, but the guidelines from the Norwegian Health Directory [21] are in line with the international guidelines [6].

Study assessment visits

We will see women for research visits at baseline (12-16 weeks of pregnancy), and again in week 37 (range 36-38), as well at three months post partum (Figure 2). We also obtain clinical measures that are collected during the delivery hospitalization as well as through primary care.

Primary outcome measure

The primary outcome measure is gestational weight gain, calculated as the difference between weight mea-sured at the time of inclusion and weight just before delivery. We measure maternal body weight at enrol-ment and before delivery to the nearest 0.1 kg with a calibrated electronic scale (SECA 770, Medema, Nor-way) with participants wearing indoor clothing, without shoes. Hospital staff will measure weight during the delivery hospitalization using a different scale than at baseline. We will calibrate the scales to ensure compar-ability. If the hospital staff forget to weight the women, we will use their self-reported weight at the time of delivery as the outcome measure.

Secondary outcome measures

The maternal secondary outcome measurements are outlined in Figure 2 and described more in detail below.

Blood sampling for fasting glucose concentrations and the other blood markers will be taken after a 10 hour overnight fast, and glucose tolerance will be measured by a 2 hour 75 mg per-oral glucose tolerance test. Gestational diabetes is diagnosed as fasting glucose ≥ 6.9 mmol/L or 2 h concentration ≥ 7.8 mmol/L [22]. Insulin resistance will be calculated using the homeosta-sis model assessment (HOMA-IR). We will analyse fast-ing blood for concentrations of lipids, ferritin, haemoglobin, high-sensitive C-reactive protein, and insulin c-peptide. We will also collect whole blood and serum to be frozen at -80C and stored in a biobank for

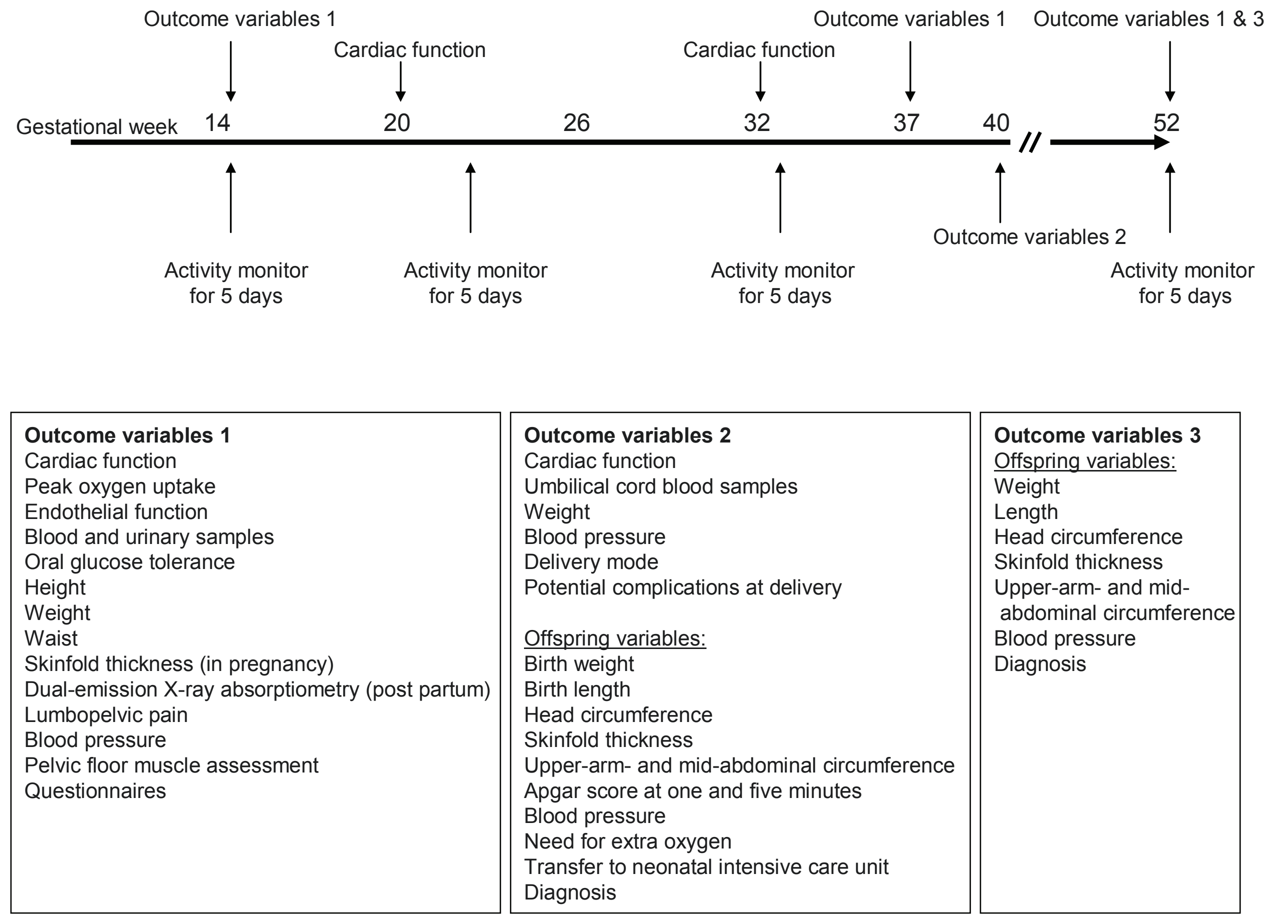

Figure 2 Outcome variables and time-points of assessment.

later analyses of hormones associated with female reproduction and blood markers associated with adiposity and insulin resistance. We will also collect Tempus blood RNA tubes and urinarysamples to be frozen for later analyses.

Body height will be measured by a wall mounted height scale. Body composition will be assessed by skinfold thickness during pregnancy and also by dual energy x-ray absorptiometry (DEXA-scan) at three months postpartum. Skinfold thickness will be measured at the right side of the body at the following sites: triceps, biceps, and subscapular, by Harpenden Caliper (Holtain Ltd, UK). Sum of skinfold thickness are measured and used for later calculation of body fat percentage. Waist circumference is measured at all time points at the level of the umbilicus. DEXA (Hologic Discovery-A: Integrity Medical Systems) will be used to measure body composition after 10 hours of fasting, to decrease large variations in hydration status (postpartum only).

Lumbopelvic pain (Disability Rating Index) and physical examination of lumbar spine and pelvic region are done by experienced physical therapists. Tests used are active straight leg raising [23] and the posterior pelvic pain provocation test [24].

Prevalence and severity of urinary- and fecal incontinence will be assessed by questionnaires [25,26], muscle strength measurements, 2D and 3D ultrasound investigation of the pelvic muscles, and clinical examination and palpation of the pelvic floor muscles. Pelvic floor muscle strength, and vaginal squeeze pressure, will be measured using a vaginal balloon catheter with a balloon size of 6.7 × 1.7 cm connected to a pressure transducer [27].

Psychological well-being and postnatal depression will be assessed using standardized questionnaires (The Psychological General Well-Being inventory [28] and The Edinburgh Postnatal Depression Scale [29], respectively). Also, the women will fill in a questionnaire about delivery expectancy (The Wijma Delivery Expectancy/Experience Questionnaire [30]) Quality of life will be assessed by the generic SF-8 Quality of life questionnaire [31]. To register diet during the intervention period, the women will fill in a validated Norwegian quantitative food frequency questionnaire [32].

Prevalence of pre-eclampsia will be registered by use of the women's health certificates. We will measure systolic and diastolic blood pressure with an automatic device, after 15 minutes of supine resting and use the average of three repeated measurements taken with two minutes intervals. We will measure endothelial function by flow-mediated dilatation of the brachial artery using ultrasound (Vivid 7, GE Vingmed Ultrasound, Norway). The women will fast and abstain from exercise, caffeine, and smoking for ten hours, and rest for 10 minutes before the measurements. The recordings are done 5 cm above the antecubital fossa before inflation of a pneumatic cuff on the lower arm for 250 mm Hg for five minutes, and again directly after cuff release and for five minutes. The responses will be analysed by a automatic detection program, and will be reported both as absolute changes and as responses normalized by dividing the percentage change in diameter by the shear rate.

Physical activity will be registered by questionnaires. Both groups will also wear a activity monitor (Sensewear Armband, APC Cardiovascular, UK) to register their level of daily physical activity for one week in early (before week 17), mid (week 19-24) and late (after week 28) pregnancy. This armband includes a two-axis accelerometer, a heat flux sensor, a galvanic skin response sensor, a skin temperature sensor and a near-body ambient temperature sensor, and has been validated during pregnancy (Berntsen et al, In review/in press Acta Obstetrica et Gyencologia Scandinavia). In addition, the training group will fill in a training diary.

Maternal cardiac function will be measured using echocardiography. The assessments will be done at week 14, week 20, and week 32, as well as 48 hours after delivery, and again at three months post partum (Figure 2). A full resting echocardiogram will be performed with a Vivid 7 scanner (GE Vingmed Ultrasound, Horten, Norway) using a phased-array transducer. Three cine loops from the three standard LV apical planes (four-chamber, two-chamber and long-axis), right ventricle and LV parasternal view will be recorded in B- mode and tissue Doppler mode simultaneously. Conventional Doppler flow parameters will be measured as well as tissue Doppler imaging with pulsed tissue Doppler in the AV-plane and strain/strain rate of the 16 segments of the left ventricle (with tissue Doppler and speckle tracking). For automated identification of myocardial segments and analysis, we will use a customized post-processing system (GcMat, GE-Vingmed, Horten, Norway).

Multistage submaximal exercise tests will be done on treadmills. After familiarizing with walking on a treadmill and 2-3 minutes of warming up, the test begins with walking at 4,5 km/h and 0% inclination. Each stage is 4 minutes and the inclination is elevated 3% each stage. Heart rate and oxygen uptake will be measured continuously during the test. Blood lactate, blood pressure and perceived exertion (according to the Borg 6-20 scale, [18]) will be recorded at the end of each stage. Tests are terminated if the subjects are feeling unwell (have symptoms of pain, nausea, or dizziness), if the heart rate exceeds 185 beats per minute, or if systolic and diastolic blood pressure exceeds 200 and 100 mmHg, respectively.

At delivery, we register mode of delivery and potential complications. Offspring variables include are outlined in Figure 2, and include the child's condition at birth and in the newborn period, birth weight, birth length, head circumference, subscapular and triceps skinfold thickness, upper-arm- and mid-abdominal circumferences, cord blood markers of inflammation and insulin resistance, and blood pressure. The child's condition include Apgar scores at one and five minutes as recorded by the attending mid-wife or physician, birth traumas, need for extra oxygen, transfer to neonatal intensive care unit, and diagnosis. The anthropometric measurements will be standardized according to Vik et al [33].

We intend to follow the children at age 3 and 12 months. At these time points we will also register breast feeding and the use of supplementary feeding, as well as crying behaviour according to Wessel et al [34]. Neuromotor development will be recorded through milestones, and by using the age and stages questionnaire at 12 months of age [35]. This instrument has been validated in Norway [36]. We also intend to follow these children for a longer time in order to study possible long term effects of in utero exposure to maternal exercise training.

Sample size

Based on prior studies [37,38], the power calculation has taken into account a 6 kg expected and clinically relevant difference between mean weight increases in the control group compared to the training group (between baseline and delivery). Based on this assumption, an independent samples t-test, 5% level of significance and test strength of 0.90, gives a study population of 59 in each group. A 15% estimated drop-out requires a total of 150 included obese pregnant women, 75 enrolled in each arm.

Ethical considerations

The Regional Committee for Medical Research Ethics has approved the study, and it will be conducted in accordance with the Declaration of Helsinki. All mothers will give their informed, written consent to participate.

Blinding

Baseline measurements, except for the armband registration of physical activity, will be done before randomisation. Later assessments will be done both blinded (echocardiography, pelvic floor assessments, blood analyses, all offspring variables) and non-blinded to group allocation (weight, skinfold measurements, lumbopelvic pain, oxygen uptake and endothelial function recordings). Although endothelial function recordings are done non-blinded, the analyses of these data will be done blinded to both group allocation and time of measurement.

Statistical methods

The principal analysis will be done on an intention-to-treat basis; outcome measures will be analyzed according to the treatment arm to which patients are randomized regardless of subsequent crossover or non-adherence. To model the outcome variables over time, we will use a linear mixed effects model [39]. Age, parity, and BMI will be considered as potential covariates to improve precision. We will also do post-hoc comparisons of time points within groups, looking at within-group changes in outcome variables from gestational week 14 to 37, as well as from gestational week 37 to 12 weeks post partum.

In addition to the primary analysis, we will split the women according to if they have actually been exercising during pregnancy or not. The cut-off for this analysis will be: 1) attending ≥ 42 organised exercise sessions, or 2) attending ≥ 28 exercise sessions + performing ≥ 28 home exercise sessions, or 3) performing ≥ 60 home exercise sessions. To count as a home exercise session, the exercise should be ≥ 50 minutes (either aerobic or strength training) of at least moderate intensity. We also intend to compare women fulfilling the general recommendations for healthy adults of exercising moderately for 30 minutes daily [40], with the ones below this threshold. Results will be given as mean values with 95% confidence intervals. P-values < 0.05 will be considered significant.

Discussion

Maternal obesity is regarded a high-risk obstetric condition and is associated with pregnancy complications and adverse outcomes [2]. In addition, there is increasing evidence that gestational weight gain may be an important predictor of the women's risk of subsequent obesity and diabetes. It has therefore been proposed that pregnancy is a unique period of time with regard to changing women's behaviour [41]. In the present study, we aim to prevent excess gestational weight gain and obesity related pregnancy complications through regular exercise training throughout pregnancy.

A recent study showed that approximately 60% of overweight women gain more than recommended during pregnancy, and as gestational weight gain associates with weight retained during the postpartum period [3], excess gestational weight gain could accelerate the obesity epidemic. Thus the prevention of weight gain in overweight and obese pregnant women is an important public health issue. Further, maternal obesity is associated with a number of adverse outcomes during and

after pregnancy, such as gestational diabetes, preeclampsia, caesarean delivery and children large for gestational age [2]. The risk for gestational diabetes is increased 2-3 fold with obesity, and fetuses of obese mothers have higher risk of developing insulin resistance in utero [42]. Greater maternal gestational weight gain has also repeatedly been found to associate with offspring adiposity in childhood [4] and in early adulthood [5]. As obese children have elevated levels of inflammatory markers related to cardiac disease manifested later in life [43], they will be at increased risk for subsequent cardiovascular disease. Importantly, there are indications in the recent literature that the prenatal environment plays a role for children obesity, independent of genetic predisposition and shared eating habits [44,45].

Strengths of our study include the thorough testing that will be done of the women as well as their offspring; investigating possible effects of exercise training on weight gain, endothelial function, insulin resistance, cardiac function, incontinence problems, lumbopelvic pain, and psychological wellbeing. In addition we collect comprehensive information on physical activity, using both subjective and objective measurements (questionnaire and armband activity registrations, respectively), as well as on dietary habits. Also regarded as a strength of the study, is the composition of the exercise training program, comprising both endurance training, general strength training and specific pelvic floor exercises.

A possible weakness of our study is that it could be underpowered for small differences in gestational weight gain between groups. Also, we think that women who like to participate in this kind of study are motivated for exercise and thereby that some of the women in the control group will do regular exercise training on their own. Such cross-over from the control group would potentially lead to smaller between-group differences.

The results from our study will give grounds for giving advice as well as organizing exercise training groups for women with obesity entering pregnancy. If women randomized to training manage to reduce their gestational weight gain compared to controls, such programs should be considered as part of the regular pregnancy care for this high-risk obstetric group.

In addition to effects on weight gain, we hope to see a reduction in other pregnancy complications. Our study will investigate the ability of regular exercise training to prevent gestational diabetes mellitus, as well as the effect on serum biomarkers associated with insulin resistance and inflammation. Previous work have found that exercise training may reduce lumbopelvic pain [46,47], however, a preventive effect of exercise during pregnancy remains unclear. Pregnancy and childbirth may also cause urinary and fecal incontinence, and obesity is an additional risk factor. The average prevalence of urinary incontinence during pregnancy and after delivery is 30-40%, and of fecal incontinence after delivery 4-5% [48]. Specific pelvic floor muscle exercises in pregnancy and post partum reduce urinary incontinence, while the preventive effect on fecal incontinence is less documented [49].

Although observational evidence is quite consistent regarding the association between large gestational weight gain and offspring adiposity, the evidence for causality is still lacking. It is possible that the intrauterine experience of infants born to mothers who gain a lot when pregnant programs long-term weight regulation, or the maternal weight gain could just be a marker of other, shared causes of both maternal and offspring weight [50]. The optimal way to explore the impact of the intrauterine exposure upon child adiposity, would be to randomized women to usual care or to an effective intervention limiting gestational weight gain, and then to follow the infants longitudinally. To our knowledge, no such adequately powered, randomized trial has been done to investigate the possible causality between excess gestational weight gain and child obesity. The randomized trial we propose here will provide evidence for such a causal relationship.

List of abbreviations

ETIP: Exercise training in pregnancy; BMI: Body mass index; LGA: Large for gestational age; SGA: Small for gestational age; HOMA-IR: Insulin resistance homeostasis model assessment; DEXA: dual energy x-ray absorptiometry; 2D: two-dimensional; 3D: three-dimensional; SF-8: Short form 8; LV: left ventricular; AV-plane: atrioventricular plane

Acknowledgements and Funding

This trial is funded by the Norwegian Fund for Postgraduate Training in Physiotherapy (TM), and the Norwegian Research Council (CBI), the Harvard Pilgrim Health Care Institute (EO). Study design and conduction, writing of manuscript and submission for publication is done independently of the funding body/bodies.

Author details

[1]Department of Public Health and General Practice, Norwegian University of Science and Technology, Trondheim, Norway. [2]Department of Obstetrics and Gynecology, St. Olavs Hospital, Trondheim University Hospital, Norwegian University of Science and Technology, Trondheim, Norway. [3]K.G. Jebsen Center of Exercise in Medicine, Department of Circulation and Medical Imaging, Norwegian University of Science and Technology, Trondheim, Norway. [4]Department of Laboratory Medicine, Norwegian University of Science and Technology, Trondheim, Norway. [5]Department of Population Medicine, Harvard Medical School and Harvard Pilgrim Health Care Institute, Boston MA, USA.

Authors' contributions

TMparticipated in the design of the study, coordinates the study and drafted the manuscript. KÅS participated in conceiving and designing the study and in critically revising the manuscript, as well as approving the final version to be published. CBIparticipated in conceiving and designing the study and in critically revising the manuscript, as well as approving the final version to be published. TVparticipated in conceiving and designing the study and in critically revising the manuscript, as well as approving the final version to be published. EOparticipated in conceiving and designing the study and in critically revising the manuscript, as well as approving the final version to be published. SMparticipated in conceiving and designing the study and in

critically revising the manuscript, as well as approving the final version to be published

Competing interests
The authors declare that they have no competing interests.

Received: 27 January 2011 Accepted: 17 June 2011
Published: 17 June 2011

References
1. Flegal KM, Carroll MD, Ogden CL, Curtin LR: **Prevalence and trends in obesity among US adults, 1999-2008.** *JAMA* 2010, **303**:235-241.
2. Baeten JM, Bukusi EA, Lambe M: **Pregnancy complications and outcomes among overweight and obese nulliparous women.** *Am J Public Health* 2001, **91**:436-440.
3. Walker LO, Sterling BS, Timmerman GM: **Retention of pregnancy-related weight in the early postpartum period: implications for women's health services.** *J Obstet Gynecol Neonatal Nurs* 2005, **34**:418-427.
4. Oken E, Taveras EM, Kleinman KP, Rich-Edwards JW, Gillman MW: **Gestational weight gain and child adiposity at age 3 years.** *Am J Obstet Gynecol* 2007, **196**:322.e321-328.
5. Mamun AA, O'Callaghan M, Callaway L, Williams G, Najman J, Lawlor DA: **Associations of gestational weight gain with offspring body mass index and blood pressure at 21 years of age: evidence from a birth cohort study.** *Circulation* 2009, **119**:1720-1727.
6. **Exercise during pregnancy and the postpartum period.** *Clin Obstet Gynecol* 2003, **46**:496-499.
7. Hinton PS, Olson CM: **Predictors of pregnancy-associated change in physical activity in a rural white population.** *Matern Child Health J* 2001, **5**:7-14.
8. Clapp JF, Dickstein S: **Endurance exercise and pregnancy outcome.** *Med Sci Sports Exerc* 1984, **16**:556-562.
9. Andreasen KR, Andersen ML, Schantz AL: **Obesity and pregnancy.** *Acta Obstet Gynecol Scand* 2004, **83**:1022-1029.
10. Cnattingius S, Bergstrom R, Lipworth L, Kramer MS: **Prepregnancy weight and the risk of adverse pregnancy outcomes.** *N Engl J Med* 1998, **338**:147-152.
11. Artal R, Catanzaro RB, Gavard JA, Mostello DJ, Friganza JC: **A lifestyle intervention of weight-gain restriction: diet and exercise in obese women with gestational diabetes mellitus.** *Appl Physiol Nutr Metab* 2007, **32**:596-601.
12. Clapp JF, Kim H, Burciu B, Schmidt S, Petry K, Lopez B: **Continuing regular exercise during pregnancy: effect of exercise volume on fetoplacental growth.** *Am J Obstet Gynecol* 2002, **186**:142-147.
13. Hopkins SA, Baldi JC, Cutfield WS, McCowan L, Hofman PL: **Exercise Training in Pregnancy Reduces Offspring Size without Changes in Maternal Insulin Sensitivity.** *J Clin Endocrinol Metab* 2010.
14. Clapp JF, Kim H, Burciu B, Lopez B: **Beginning regular exercise in early pregnancy: effect on fetoplacental growth.** *Am J Obstet Gynecol* 2000, **183**:1484-1488.
15. Garshasbi A, Faghih Zadeh S: **The effect of exercise on the intensity of low back pain in pregnant women.** *Int J Gynaecol Obstet* 2005, **88**:271-275.
16. Polley BA, Wing RR, Sims CJ: **Randomized controlled trial to prevent excessive weight gain in pregnant women.** *Int J Obes Relat Metab Disord* 2002, **26**:1494-1502.
17. Asbee SM, Jenkins TR, Butler JR, White J, Elliot M, Rutledge A: **Preventing excessive weight gain during pregnancy through dietary and lifestyle counseling: a randomized controlled trial.** *Obstet Gynecol* 2009, **113**:305-312.
18. Borg GA: **Psychophysical bases of perceived exertion.** *Med Sci Sports Exerc* 1982, **14**:377-381.
19. Hettema J, Steele J, Miller WR: **Motivational interviewing.** *Annu Rev Clin Psychol* 2005, **1**:91-111.
20. Rasmussen KM, Yaktine AL: *Weight gain during pregnancy: reexamining the guidelines* Washington, D.C.: National Academies Press; 2009.
21. Bahr R: *Aktivitetshåndboken: fysisk aktivitet i forebygging og behandling* [Oslo]: Helsedirektoratet; 2009.
22. *Definition and diagnosis of diabetes mellitus and intermediate hyperglycemia* Geneve: International diabetes federation; 2006.
23. Mens JM, Vleeming A, Snijders CJ, Koes BW, Stam HJ: **Reliability and validity of the active straight leg raise test in posterior pelvic pain since pregnancy.** *Spine (Phila Pa 1976)* 2001, **26**:1167-1171.
24. Ostgaard HC, Zetherstrom G, Roos-Hansson E, Svanberg B: **Reduction of back and posterior pelvic pain in pregnancy.** *Spine (Phila Pa 1976)* 1994, **19**:894-900.
25. Sandvik H, Hunskaar S, Vanvik A, Bratt H, Seim A, Hermstad R: **Diagnostic classification of female urinary incontinence: an epidemiological survey corrected for validity.** *J Clin Epidemiol* 1995, **48**:339-343.
26. Vaizey CJ, Carapeti E, Cahill JA, Kamm MA: **Prospective comparison of faecal incontinence grading systems.** *Gut* 1999, **44**:77-80.
27. Bo K: **Pelvic floor muscle strength and response to pelvic floor muscle training for stress urinary incontinence.** *Neurourol Urodyn* 2003, **22**:654-658.
28. Wenger NK: *Assessment of quality of life: in clinical trials of cardiovascular therapies* [S.l]: Le Jacq Publishing; 1984.
29. Cox JL, Holden JM, Sagovsky R: **Detection of postnatal depression. Development of the 10-item Edinburgh Postnatal Depression Scale.** *Br J Psychiatry* 1987, **150**:782-786.
30. Wijma K, Wijma B, Zar M: **Psychometric aspects of the W-DEQ; a new questionnaire for the measurement of fear of childbirth.** *J Psychosom Obstet Gynaecol* 1998, **19**:84-97.
31. Ware JE: *How to score and interpret single-item health status measures: a manual for users of the SF-8 Health Survey* Lincoln, RI: QualityMetric; 2001.
32. Nes M, Frost Andersen L, Solvoll K, Sandstad B, Hustvedt BE, Lovo A, Drevon CA: **Accuracy of a quantitative food frequency questionnaire applied in elderly Norwegian women.** *Eur J Clin Nutr* 1992, **46**:809-821.
33. Vik T, Markestad T, Ahlsten G, Gebre-Medhin M, Jacobsen G, Hoffman HJ, Bakketeig LS: **Body proportions and early neonatal morbidity in small-forgestational-age infants of successive births.** *Acta Obstet Gynecol Scand Suppl* 1997, **165**:76-81.
34. Wessel MA, Cobb JC, Jackson EB, Harris GS Jr, Detwiler AC: **Paroxysmal fussing in infancy, sometimes called colic.** *Pediatrics* 1954, **14**:421-435.
35. Squires J, Bricker D, Potter L: *The ASQ user's guide* Baltimore: Paul H. Brookes; 1999.
36. Richter J, Janson H: **A validation study of the Norwegian version of the Ages and Stages Questionnaires.** *Acta Paediatr* 2007, **96**:748-752.
37. Christiansen T, Paulsen SK, Bruun JM, Pedersen SB, Richelsen B: **Exercise training versus diet-induced weight-loss on metabolic risk factors and inflammatory markers in obese subjects: a 12-week randomized intervention study.** *Am J Physiol Endocrinol Metab* 2010, **298**:E824-831.
38. Wolff S, Legarth J, Vangsgaard K, Toubro S, Astrup A: **A randomized trial of the effects of dietary counseling on gestational weight gain and glucose metabolism in obese pregnant women.** *Int J Obes (Lond)* 2008, **32**:495-501.16.
39. Pinheiro JC, Bates DM: *Mixed-effects models in S and S-PLUS* New York: Springer; 2000.
40. Haskell WL, Lee IM, Pate RR, Powell KE, Blair SN, Franklin BA, Macera CA, Heath GW, Thompson PD, Bauman A: **Physical activity and public health: updated recommendation for adults from the American College of Sports Medicine and the American Heart Association.** *Circulation* 2007, **116**:1081-1093.
41. Lawlor DA, Chaturvedi N: **Treatment and prevention of obesity–are there critical periods for intervention?** *Int J Epidemiol* 2006, **35**:3-9.
42. Catalano PM, Presley L, Minium J, Hauguel-de Mouzon S: **Fetuses of obese mothers develop insulin resistance in utero.** *Diabetes Care* 2009, **32**:1076-1080.
43. Skinner AC, Steiner MJ, Henderson FW, Perrin EM: **Multiple markers of inflammation and weight status: cross-sectional analyses throughout childhood.** *Pediatrics* 2010, **125**:e801-809.
44. Smith J, Cianflone K, Biron S, Hould FS, Lebel S, Marceau S, Lescelleur O, Biertho L, Simard S, Kral JG, Marceau P: **Effects of maternal surgical weight loss in mothers on intergenerational transmission of obesity.** *J Clin Endocrinol Metab* 2009, **94**:4275-4283.
45. Kral JG, Biron S, Simard S, Hould FS, Lebel S, Marceau S, Marceau P: **Large maternal weight loss from obesity surgery prevents transmission of obesity to children who were followed for 2 to 18 years.** *Pediatrics* 2006, **118**:e1644-1649.
46. Elden H, Ladfors L, Olsen MF, Ostgaard HC, Hagberg H: **Effects of acupuncture and stabilising exercises as adjunct to standard treatment**

in pregnant women with pelvic girdle pain: randomised single blind controlled trial. *BMJ* 2005, **330**:761.
47. Morkved S, Salvesen KA, Schei B, Lydersen S, Bo K: **Does group training during pregnancy prevent lumbopelvic pain? A randomized clinical trial.** *Acta Obstet Gynecol Scand* 2007, **86**:276-282.
48. Morkved S, Bo K: **Prevalence of urinary incontinence during pregnancy and postpartum.** *Int Urogynecol J Pelvic Floor Dysfunct* 1999, **10**:394-398.
49. Mørkved S: **Evidence for pelvic floor physiotherapy for urinary incontinence during pregnancy and after childbirth.** In *Evidence based physiotherapy for the pelvic floor: Bridging research and clinical practice.* Edited by: Bø K, Bermans B, Van Kampen M, Mørkved S. Elsevier; 2007:.
50. Oken E: **Maternal and child obesity: the causal link.** *Obstet Gynecol Clin North Am* 2009, **36**:361-377, ix-x.

doi:10.1186/1745-6215-12-154
Cite this article as: Moholdt *et al.*: Exercise Training in Pregnancy for obese women (ETIP): study protocol for a randomised controlled trial. *Trials* 2011 **12**:154.

Submit your next manuscript to BioMed Central and take full advantage of:

- **Convenient online submission**
- **Thorough peer review**
- **No space constraints or color figure charges**
- **Immediate publication on acceptance**
- **Inclusion in PubMed, CAS, Scopus and Google Scholar**
- **Research which is freely available for redistribution**

Submit your manuscript at
www.biomedcentral.com/submit

BioMed Central
