# Supplementary material for: Cardiac function in newborns of obese women and the effect of exercise during pregnancy. A randomized controlled trial
Source: PLoS One. 2018 Jun 1;13(6):e0197334. doi: 10.1371/journal.pone.0197334 (PMC5983429; doi:10.1371/journal.pone.0197334)
Supplement: S3 Protocol — (PDF) [file pone.0197334.s003.pdf]

Exercise Training in Pregnancy. Good for obese women and their fetus/newborn?

Background and status of knowledge

Pregnancy and the postpartum period are risk periods for development of excessive weight gain, gestational diabetes, urinary and fecal incontinence and musculo-skeletal problems such as lumbopelvic pain [1, 2]. Exercise is recommended to reduce negative physical and psychological symptoms [3, 4].

Exercise is defined as regular, leisure-time physical activities, to improve physical condition, ability or health. Thus, exercise must have a high enough level of intensity and regularity to improve the function of the heart and respiratory systems and muscles. Most published studies indicate that women with normal, uncomplicated pregnancies are fit for exercise with few restrictions, without any risk of harm to the mother or fetus [1].

Observational studies demonstrate that overweight in pregnancy is a risk factor for adverse pregnancy outcomes as fetal macrosomia, prolonged labor, low Apgar score, shoulder dystocia, nerve plexus injuries, increased proportion of instrumental deliveries and perineal ruptures [5]. There is a 2.6 fold risk for gestational diabetes mellitus (fourfold in morbidly obese women) and a recent study has shown that fetuses of obese mothers develop insulin resistance in uterus [6]. Infant congenital heart defects increase with BMI>29 in early pregnancy [7] . Reduced cardiac function is seen in almost 50% of the obese subjects [8]. Weight increase > 15 kilos implies a higher risk of developing diseases in pregnancy and complications during labor [9], as well as problems with weight reduction after birth [10]. A recent review suggest that women who exercise before and during pregnancy weigh less and are less likely to gain excessive weight, while other studies conclude differently perhaps due to the difficulty of assessing the effect of exercise alone, since weight gain also depends on food intake and other factors [1, 11].

Nearly 50% of Norwegian women report lumbopelvic pain during pregnancy [12]. About 1 in 5 of women with postpartum lumbopelvic pain gets chronic pain [13]. Exercise may have a positive effect on lumbopelvic pain during pregnancy [14, 15] and after delivery [16]. However, a preventive effect of exercise during pregnancy remains unclear.

Pregnancy and childbirth may cause urinary and fecal incontinence, and overweight is an additional risk factor. Average prevalence of urinary incontinence during pregnancy and after delivery are 30-40%, and of fecal incontinence after delivery 4-5% [2]. Specific pelvic floor muscle exercises in pregnancy and post partum reduce urinary incontinence, while the preventive effect and effects on fecal incontinence is less documented [17].

Today's knowledge about the importance of regular exercise in pregnancy is mainly based on results from observational studies [18-22]. There is a lack of results from RCT's with high methodological quality, assessing short and long term effects of exercise in pregnancy on mothers and offspring.

Aims of the study

Main aims of this study are to assess if regular exercise in pregnancy among overweight and obese women can prevent or influence

- weight gain
- insulin resistance/sensitivity
- lumbopelvic pain
- urinary and/or fecal incontinence
- prolonged labor and other complications
- impaired cardiac function in mother and fetus/newborn

Methods

Design

A RCT where the women are randomly allocated (following a computerized randomization procedure) to training and control groups. Measurements are carried out with validated instruments before and after the training period, at labor (weight and cardiac function), one week (weight and cardiac function) and 3 months after delivery.

Subjects

Pregnant women with pre-pregnancy BMI ≥ 28 are invited to participate in the trial. Women are eligible if they are 18 years or older, with a singleton live fetus at the routine ultrasound

scan. Exclusion criteria are pregnancy complications, high risk for preterm labor or diseases that could interfere with participation. Recruitment strategies: The women are given detailed information about the project at the ordinary visits at general practitioners and midwives and at outpatient clinics at the hospitals. Also, participants in another study at the hospital (regarding early ultrasound in week 12 and pre-eclampsia) will get information about the present study. We will also include 20 women with a BMI below 25 to serve as a control group regarding cardiac function (for mother and fetus/child) and endothelial function. These women will not be randomised to training or not, and will not undergo other tests except those on flow-mediated dilatation and cardiac function.

Intervention

Standard dietary advice is given to all women. The training group follows a specially designed exercise program including aerobic activity, specific exercises for stabilization of the lower back and pelvis, and the pelvic floor muscles. They attend training groups for a total of 60 minutes 2 (-4) times weekly between 14 and 34 pregnancy weeks. The women are also encouraged to come for exercise training in the weeks after week 34 if they feel like. All women will have to come to exercise training for a minimum of 2 times weekly, and have the opportunity to attend as much as 4 times per week if they like. The endurance training consists of walking on treadmills for 25 minutes after warming up in 10 minutes. The intensity will be moderate, reaching ~80% of their maximal capacity in periods (corresponding to Borg scale12-15). In addition, they follow a 50 minutes home exercise program at least once per week (35 minutes endurance training and 15 minutes strength exercises) as well as doing daily pelvic floor muscle exercises. They are also recommended to be physically active in addition to the training program. Adherence is strongly emphasized and registered in the women's personal training diary and the reports from the persons leading the training groups. The training protocol follows recommendations from Helsedirektoratet 2009 [4] and ACOG 2003 [3]. Specific adjustments are made according to aerobic activities if needed (for example using a stationary bike instead of treadmill walking). During the training period, the subjects will go through motivational interviews in a group setting. Women in the control group will receive the customary regular consultations with midwife, general practitioner and obstetrician. They are not discouraged from exercising on their own.

Outcome measures

(at 14 and 34 weeks of pregnancy and 3 months post partum)

Primary outcome measure is weight increase based on weight measured at 14 weeks and immediately before labor.

Secondary outcome measures are:

- Insulin resistance and gestational diabetes: Peroral glucose tolerance tests (OGTT) are done at 14 and 34 weeks of pregnancy and 3 months post partum. Fasting and 2 hours glucose level are used. HOMA-IR is measured at 14 and 34 weeks of pregnancy and 3 months post partum. Gestational diabetes is diagnosed according to standard criteria.
- Weight, Body Mass Index (BMI), skin-fold thickness (subscapular and triceps skinfold thickness measurements by Harpenden Caliper, sum of subscapular and triceps skinfold thicknesses and the ratio between these; S-T- ratio). Waist circumference is measured at all time points at the level of the umbilicus. In addition, Dual Energy X-ray absorptiometry (dexa scan) is done at 3 months post partum.
- Levels of hormones associated with female reproduction and hormones and other factors associated with adiposity and insulin resistance will be investigated in blood samples (full blood and serum are collected following standardized procedures, frozen and stored in a biobank). Urinary samples are taken to assess if pre-eclampsia can be detected at an earlier stage than when using traditional markers.
- Lumbopelvic pain (Disability Rating Index) and physical examination of lumal spine and pelvic region.
- Urinary- and fecal incontinence (stress-test, muscle strength measurements, ultrasound of the pelvic muscles, using 2D and 3D ultrasound, Urinary severity index, St.Marks fecal incontinence score, Clinical examination and palpation of the pelvic floor muscles: Vaginal palpation and observation during contraction to assess the women’s ability to perform pelvic floor muscle contraction. Pelvic floor muscle strength, vagnial squeeze pressure, using a vaginal balloon catheter with a balloon size of 6.7 x 1.7 cm connected to a pressure transducer [23])
- Psychological well-being (Psychological General Well-Being inventory, PGWB)
- Postnatal depression (The Edinburgh Postnatal Depression Scale)
- Delivery expectancy (The Delivery Expectancy/Experience Questionnaire, W-DEQ)
- Quality of life (generic) (SF-8)
- Nutrition (Norkost - a quantitative food frequency questionnaire)
- Blood pressure (systolic-, diastolic- and mean, using an automated, standardized method)

- Physical activity (Questionnaire and Armband registration of movement, Borg's Rating Scale of Perceived Exertion)
- Cardiac function of mother and fetus/newborn using echocardiography, measured at 14, 18, and 34 weeks, as well as directly after labour and in the 1 week old infant.
- Endothelial function (Flow mediated dilatation of the brachial artery using ultrasound), measured at 14 and 34 weeks, and at 3 months post partum
- Peak oxygen uptake, measured at 14 and 34 weeks, and at 3 months post partum.

Power calculation

The power calculation has taken into account a 6 kg expected and clinical relevant difference between mean weight increases* in the control group compared to the training group (between 14 weeks of pregnancy and labour) [24, 25]. Based on this assumption an independent samples t-test, 5% level of significance and test strength of 0.90 give a study population of 59 in each group. A 15% estimated drop-out requires a total of 150 included overweight/obese pregnant women.

Statistical analyses

The principal analysis will be done on an intention-to-treat basis. Additional subgroup analyses will be carried out. Results will be given as mean values with 95% confidence intervals (CI). P-values < 0.05 will be considered significant.

Ethical considerations

The procedures followed will be in accordance with the ethical standards of the responsible regional committee on human experimentation and with the Helsinki declaration.

Main activities and milestones in the project period

**01.07.10 – 31.05.13**	**2010**				**2011**				**2012**			
	**1**	**2**	**3**	**4**	**1**	**2**	**3**	**4**	**1**	**2**	**3**	**4**
Preparations/Post doc appointment	x	x										
Inclusion/intervention			x	x	x	x	x	x	x	x		
Data collection/statistical		x	x	x	x	x	x	x	x	X	x	x

analysis												
Publication 1,2 and 3						x	x	x	x	x	x	
Publication 4, 5 and 6									x	x	x	X

Perspectives and compliance with strategic documents

Compliance with strategic documents

This proposal addresses several aspects of the St.Olavs Hospital's strategic goals, including clinical research in women's health. Moreover, it represents translational research as it combines competence in the basic sciences and laboratory studies with clinical research, aiming at providing better evidence for pregnancy care.

Relevance to society

In both national and international literature the importance of physical activity are highlighted. WHO has presented a global strategy for nutrition, physical activity and health (Sosial-og helsedepartementet, 2004). In Norway physical activity and exercise have been strongly addressed also in pregnant and postpartum women, and research in women's health is an issue of high priority (St.meld.nr.16, Handlingsplan for fysisk aktivitet and Nasjonal Helseplan 2007-2010). Following a recent review from leading experts in women's health; ***"prospective clinical trials to establish the efficacy of exercise for weight-gain restriction during pregnancy with resulting lower risk of maternal and fetal co-morbidities are perhaps the greatest research need"*** . The research topic has clearly global relevance.

Project management, organization and cooperation

The project managers have broad experience in physiotherapy, exercise physiology, obstetrics and gynaecology, and pediatrics, and all three are experienced in working with data from large clinical trials and population based studies and as mentors for a number of students at different levels.

Siv Mørkved is a physiotherapist and professor at the Faculty of Medicine, NTNU. Her main field of research has been in women's health. Most of her publications are related to effects of interventions to treat pelvic floor dysfunction and pregnancy-related diseases. Dr. Mørkved has initiated and been the project manager of a number of RCT's in different patient groups at St. Olav University Hospital. Thus, she has already documented significant experience and competence in conducting RCT's within a field much more complicated than studies on the effects of drugs. She is co-mentor for two and principal mentor for three PhD candidates. Dr Mørkved initiated and designed the ongoing RCT "Regular exercise during pregnancy", and is principal investigator of this proposal.

Kjell Å. Salvesen is professor of obstetrics and gynecology at NTNU. He is consultant at the National Center for Fetal Medicine, St. Olav University Hospital, and vice-chair at the Department of Laboratory Medicine, Women's and Children's Health. His main field of research has been within fetal medicine, safety aspects of ultrasound in pregnancy, pre-eclampsia, post-term births and physical activity in pregnancy. He has published more than 80 papers, 40 of these during the last 5 years. He has been principal or co-mentor of seven PhD candidates. He is currently mentor of five other candidates.

Torstein Vik is professor of paediatric and perinatal epidemiology. His thesis was on long term consequences of IUGR, in particular of growth, mental and motor development as well as on physical health. This study was population based multicenter sponsored by the National Institute of Child Health and Development, Bethesda, MD, USA. Vik has also been significantly involved in long term neuro- psychiatric consequences of children, adolescents and young adults who had low birth weight. Vik has published more than 80 papers on various pediatric topics, 25 of these during the last five years. In a number of papers dr. Vik has also addressed the early origin of adult disease hypothesis. He has been mentor of four PhD candidates, and is currently mentor of six other candidates.

Other participants in our research group are professor of endocrinology **Sven Carlsen**, associate professor **Pål Romundstad** (statistics), post doc/PT **Trine Moholdt** and professor **Ulrik Wisløff** and researcher **Charlotte Björk Ingul** at the Department of Circulation and Medical Imaging, Centre for sports and physical activity research, NTNU.

National collaboration is established with PT and professor in exercise physiology **Kari Bø**, Norwegian School of Sport Sciences and Akershus University Hospital. Professor Bø is an internationally highly respected researcher and opinion leader in women's health, and has worked with Mørkved in several projects.

International collaboration has already been established with a leading research group at Harvard Medical School, Boston, USA and with Karolinska Institutet, Stockholm, Sweden. The principal investigator has visited Dr. Oken at Harvard Medical School, discussed the project and has planned meetings during the project period (see budget). The group from Karolinska Institutet visited Trondheim in January 2009 and has together with Mørkved designed a similar study that will be carried out in Stockholm. Agreements for cooperation both from Harvard (also including guest invitation for Mørkved) and Karolinska are attached.

Dr. Emily Oken is an assistant professor in the Department of Ambulatory Care and Prevention at Harvard Medical School and Harvard Pilgrim Health Care. She has clinical training in both Internal Medicine and Pediatrics. She has extensive experience in performing observational studies regarding associations of maternal diet, physical activity, and other behaviors with both maternal and infant health. For example, she has studied associations of maternal gestational weight gain with child obesity risk, diet during pregnancy with risk for gestational diabetes, physical activity before and during pregnancy with risk for gestational diabetes mellitus, and maternal diet and physical activity with postpartum weight retention. In parallel with Dr. Mørkved, she is planning an intervention study in Boston to prevent excessive gestational weight gain among overweight women.

Dr. Lena Nilsson-Wikmar is an associate professor at Karolinska Institutet. She has conducted several studies on low back and pelvic girdle pain in pregnancy and post partum. The aim of this project is to evaluate a community based programme in order to support pregnant and postpartum women to initiate or maintain a healthy lifestyle including physical activity and behaviour change.

References

1. Gavard, JA and R Artal, Effect of exercise on pregnancy outcome. *Clin Obstet Gynecol* 2008; **51**:467-80.
2. Morkved, S and K Bo, Prevalence of urinary incontinence during pregnancy and postpartum. *Int Urogynecol J Pelvic Floor Dysfunct* 1999; **10**:394-8.
3. Exercise during pregnancy and the postpartum period. *Clin Obstet Gynecol* 2003; **46**:496-9.
4. Bahr, R, *Aktivitetshåndboken: fysisk aktivitet i forebygging og behandling*. 2009, [Oslo]: Helsedirektoratet. 624 s.
5. Baeten, JM, EA Bukusi, and M Lambe, Pregnancy complications and outcomes among overweight and obese nulliparous women. *Am J Public Health* 2001; **91**:436-40.
6. Catalano, PM, L Presley, J Minium, and S Hauguel-de Mouzon, Fetuses of obese mothers develop insulin resistance in utero. *Diabetes Care* 2009; **32**:1076-80.

7. Leddy, MA, ML Power, and J Schulkin, The impact of maternal obesity on maternal and fetal health. *Rev Obstet Gynecol* 2008; **1**:170-8.
8. Kosmala, W, C Wong, J Kuliczkowska, R Leano, M Przewlocka-Kosmala, and TH Marwick, Use of body weight and insulin resistance to select obese patients for echocardiographic assessment of subclinical left ventricular dysfunction. *Am J Cardiol* 2008; **101**:1334-40.
9. Clapp, JF, 3rd and KD Little, Effect of recreational exercise on pregnancy weight gain and subcutaneous fat deposition. *Med Sci Sports Exerc* 1995; **27**:170-7.
10. Oken, E, EM Taveras, FA Popoola, JW Rich-Edwards, and MW Gillman, Television, walking, and diet: associations with postpartum weight retention. *Am J Prev Med* 2007; **32**:305-11.
11. Rössner, S, *Physical activity and prevention and treatment of weight gain associated with pregnancy: current evidence and research issues.*, in *Physical activity and obesity*, C. Bouchard, Editor. 2000, Human Kinetics: Champaign.
12. Endresen, EH, Pelvic pain and low back pain in pregnant women--an epidemiological study. *Scand J Rheumatol* 1995; **24**:135-41.
13. Larsen, EC, C Wilken-Jensen, A Hansen, DV Jensen, S Johansen, H Minck, et al., Symptom-giving pelvic girdle relaxation in pregnancy. I: Prevalence and risk factors. *Acta Obstet Gynecol Scand* 1999; **78**:105-10.
14. Elden, H, L Ladfors, MF Olsen, HC Ostgaard, and H Hagberg, Effects of acupuncture and stabilising exercises as adjunct to standard treatment in pregnant women with pelvic girdle pain: randomised single blind controlled trial. *BMJ* 2005; **330**:761.
15. Morkved, S, KA Salvesen, B Schei, S Lydersen, and K Bo, Does group training during pregnancy prevent lumbopelvic pain? A randomized clinical trial. *Acta Obstet Gynecol Scand* 2007; **86**:276-82.
16. Stuge, B, E Laerum, G Kirkesola, and N Vollestad, The efficacy of a treatment program focusing on specific stabilizing exercises for pelvic girdle pain after pregnancy: a randomized controlled trial. *Spine (Phila Pa 1976)* 2004; **29**:351-9.
17. Mørkved, S, *Evidence for pelvic floor physiotherapy for urinary incontinence during pregnancy and after childbirth.*, in *Evidence based physiotherapy for the pelvic floor: Bridging research and clinical practice*, K. Bø, Bermans, B., Van Kampen, M., Mørkved, S., Editor. 2007, Elsevier.
18. Clapp, JF, 3rd and S Dickstein, Endurance exercise and pregnancy outcome. *Med Sci Sports Exerc* 1984; **16**:556-62.
19. Oken, E, EM Taveras, KP Kleinman, JW Rich-Edwards, and MW Gillman, Gestational weight gain and child adiposity at age 3 years. *Am J Obstet Gynecol* 2007; **196**:322 e1-8.
20. Andreasen, KR, ML Andersen, and AL Schantz, Obesity and pregnancy. *Acta Obstet Gynecol Scand* 2004; **83**:1022-9.
21. Cnattingius, S, R Bergstrom, L Lipworth, and MS Kramer, Prepregnancy weight and the risk of adverse pregnancy outcomes. *N Engl J Med* 1998; **338**:147-52.
22. Artal, R, RB Catanzaro, JA Gavard, DJ Mostello, and JC Friganza, A lifestyle intervention of weight-gain restriction: diet and exercise in obese women with gestational diabetes mellitus. *Appl Physiol Nutr Metab* 2007; **32**:596-601.
23. Bo, K, Pelvic floor muscle strength and response to pelvic floor muscle training for stress urinary incontinence. *Neurourol Urodyn* 2003; **22**:654-8.
24. Christiansen, T, SK Paulsen, JM Bruun, SB Pedersen, and B Richelsen, Exercise training versus diet-induced weight-loss on metabolic risk factors and inflammatory markers in obese subjects: a 12-week randomized intervention study. *Am J Physiol Endocrinol Metab* 2010; **298**:E824-31.

25. Wolff, S, J Legarth, K Vangsgaard, S Toubro, and A Astrup, A randomized trial of the effects of dietary counseling on gestational weight gain and glucose metabolism in obese pregnant women. *Int J Obes (Lond)* 2008; **32**:495-501.
