# Supplementary material for: Cardiac function in newborns of obese women and the effect of exercise during pregnancy. A randomized controlled trial
Source: PLoS One. 2018 Jun 1;13(6):e0197334. doi: 10.1371/journal.pone.0197334 (PMC5983429; doi:10.1371/journal.pone.0197334)
Supplement: S3 Text — Approval of the change request regarding the NeoETIP study. (PDF) [file pone.0197334.s006.pdf]

Emne: REK Midt 2010/1522 Godkjenning av endret informasjonsskriv
Fra: post@helseforskning.etikkom.no
Dato: 15.04.2011 12:55
Til: charlotte.b.ingul@ntnu.no
Kopi: trine.moholdt@ntnu.no; rek-4@medisin.ntnu.no;

Vår ref. nr.: 2010/1522
Prosjekttittel: "Trening av overvektige gravide"
Prosjektleder: Trine Moholdt

Kjære Trine Moholdt og Charlotte B. Ingul,

Vi viser til revidert informasjonsskriv innsendt på e-post 12.04.2011. Vi godkjenner endringene.

Vi ber om at alle henvendelser sendes inn via vår saksportal: http://helseforskning.etikkom.no
eller på e-post til: post@helseforskning.etikkom.no.

Vennligst oppgi vårt referansenummer i korrespondansen.

Med vennlig hilsen | Best regards

Sven Erik Gisvold
Professor dr.med
Leder REK Midt

Anneli Pellerud
Førstekonsulent
post@helseforskning.etikkom.no
T: 73597509

Regional komité for medisinsk og helsefaglig
forskningsetikk REK midt-Norge (REK midt)
http://www.helseforsking.etikkom.no

REK
REGIONALE KOMITEER FOR MEDISINSK OG HELSEFAGLIG FORSKNINGSETIKK
