# Supplementary material for: Cardiac function in newborns of obese women and the effect of exercise during pregnancy. A randomized controlled trial
Source: PLoS One. 2018 Jun 1;13(6):e0197334. doi: 10.1371/journal.pone.0197334 (PMC5983429; doi:10.1371/journal.pone.0197334)
Supplement: S4 Text — (PDF) [file pone.0197334.s007.pdf]

Forespørsel om å delta i en vitenskapelig undersøkelse

# TRENING I SVANGERSKAPET

## En randomisert klinisk studie av trening av overvektige gravide

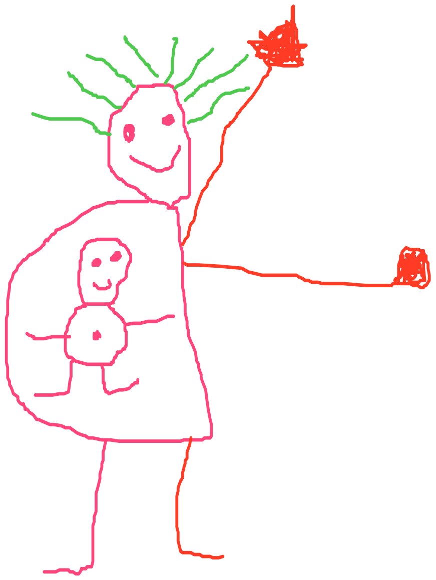

Studien er et samarbeidsprosjekt mellom NTNU og Kvinneklinikken ved St.Olavs Hospital

NTNU
Det skapende universitet

HELSE MIDT-NORGE

**Bakgrunn og målsetting**
I perioden 2010-2012 gjennomføres et forskningsprosjekt ved St. Olavs Hospital, med målsettingen å øke kunnskapen om helsen hos overvektige gravide. Undersøkelsen tar sikte på å finne ut mer om effekter av trening under svangerskapet, i forebygging og behandling av svangerskapsrelatert sykdom (svangerskapsdiabetes, høyt blodtrykk, rygg- og/eller bekkenrelaterte smerter, urin- og/eller avføringslekkasje). Vi vil også se om trening påvirker mors og fosterets hjertefunksjon i graviditeten. Dessuten vil vi undersøke om treningen påvirker fødselen.

**Hvem kan delta, og hva innebærer deltakelse**
Gravide kvinner (≥18 år) med kroppsmasseindeks* ≥ 28 inviteres til å delta. For at vi skal få vite mer om helsetilstanden generelt, og om helsen i svangerskapet spesielt, ber vi om å få ta blodprøver av alle deltakerne i prosjektet, og at alle svarer på spørreskjema og gjennomfører enkelte tester. Testingen foregår ved St.Olavs Hospital hovedsakelig i svangerskapsuke 14 (12-16) og 37, samt tre måneder etter fødselen. Testingen vil foregå over to dager, og den ene dagen må du være på sykehuset i ca tre timer fordi det skal gjøres en sukkerbelastningstest, der blodprøvene skal tas med to timers mellomrom. Du møter fastende denne dagen og vil bli tilbudt mat etterpå. Vi vil også se på hjertet og blodårene dine med ultralyd, registrere vekt og gjøre målinger av kroppssammensetning, teste utholdenhet (kondisjon), og gjøre noen tester av bekkenbunns-funksjon og bekkensmerter. Dessuten ber vi om at vi får benytte informasjon om vekt og blodtrykk fra svangerskapsjournalen, og opplysninger om fødselsforløpet og barnets vekt, lengde samt rutine barnelegeundersøkelse fra fødselsjournalen. Vi ber også om å få ta blodprøve fra navlestrengen like etter fødsel. Barnets hjertefunksjon vil bli undersøkt litt nøyere enn ved vanlig oppfølging, ved ultralyd i uke 14, 20 og 32. I tillegg vil barnet bli hjerteundersøkt mens mor og barn er innlagt på barselavdelingen, samt 6-8 uker etter hjemreise. På barselavdelingen vil også barnets kroppssammensetning beregnes.

Når det gjelder trening under svangerskapet, vet man ennå ikke sikkert om svangerskapsproblemer kan behandles og forebygges, og eventuelt hvilke tiltak som har best effekt. Deltakerne i denne undersøkelsen blir derfor tilfeldig fordelt i to grupper. En datamaskin avgjør ved loddtrekning (randomisering) hvilken behandling du skal ha. Du får et tilfeldig nummer i databasen, og bare prosjektkoordinator kjenner koblingen mellom nummeret i databasen og din identitet. Deltakerne i den ene gruppen får den informasjonen som nå gis rutinemessig til gravide. Den andre gruppen er en treningsgruppe, hvor deltakerne skal delta i en times trening to-tre ganger i uka ved St.Olavs hospital. I tillegg skal et hjemmetreningsprogram på 45 minutter gjennomføres minst en gang i uka. Treningen gjennomføres fra oppstart i studien og til tester i uke 37, eventuelt helt til fødsel hvis du orker. Treningen tilpasses din fysiske form.

*
**Kroppsmasseindeks beregnes ved:**

$$\frac{\text{Vekt (kg)}}{\text{Høyde (m) x høyde (m)}}$$

Eksempel: 84kg/1,65 m x 1,65 m = 31

Du må kunne møte til testing på dagtid. Hvis du kommer i treningsgruppa, kan du få velge mellom trening på dag- eller kveldstid. Alle deltakerne vil få en pakke med barnemat til en verdi av 500 kroner som takk for at de deltar.

Etter at forskningsprosjektet er avsluttet, vil alle deltakere få skriftlig informasjon om resultatene. Hvis trening viser seg å ha god effekt som behandling og forebygging, vil deltakerne i kontrollgruppen få informasjon om treningsprogrammet etter at prosjektet er avsluttet. For å kunne undersøke langtidsvirkningen av trening under svangerskapet, ber vi om samtykke til at data oppbevares i 20 år, slik at vi kan kontakte deltakerne igjen for eventuelle oppfølgingsstudier på din og barnets helse i årene etter fødselen.

**Frivillighet og samtykke**

- Deltakelse i prosjektet er frivillig.
- Alle deltakere i prosjektet har rett til å trekke seg fra prosjektet når de måtte ønske, uten at dette får konsekvenser for videre oppfølging og behandling. All informasjon deltakerne gir i forbindelse med prosjektet, behandles konfidensielt, og data avidentifiseres. Alle som skal ha kontakt med de innsamlede data, er underlagt taushetsplikt i henhold til Forvaltningslovens § 13 og Helsepersonellovens § 21.
- Deltakerne er dekket av Pasientskadeerstatningsordningen.

**Etisk og faglig vurdering**

- Prosjektet er vurdert av Regional komite for medisinsk forskningsetikk, Region Midt-Norge, og komiteen har godkjent at prosjektet gjennomføres.

Ansvarlige prosjektledere er Kjell Åsmund Salvesen, overlege ved Kvinneklinikken, St. Olavs Hospital, Siv Mørkved, Forskningssjef ved St. Olavs hospital, og Trine Moholdt, post doktor ved Institutt for samfunnsmedisin, NTNU.

HVIS DU ØNSKER Å DELTA, ELLER HAR SPØRSMÅL OM PROSJEKTET, BES DU KONTAKTE:

Trine Moholdt (prosjektleder)

Du kan ta kontakt på følgende måte:

- E-post: trine.moholdt@ntnu.no
- Telefon: 97098594

ID-nummer:

*(fylles ut av prosjektkoordinator)*

**Hvis du ønsker å delta må du fylle ut dette samtykkeformularet. Samtykkeformularet leveres til prosjektkoordinator ved oppmøte for første test.**

## SAMTYKKEERKLÆRING FOR PROSJEKTET ”TRENING I SVANGERSKAPET”

Jeg har lest informasjonsskrivet og har hatt anledning til å stille spørsmål. Jeg er også informert om at journalopplysninger fra det aktuelle svangerskap og fødsel vil bli gjennomgått og registrert og samtykker i å delta i studien.

Sted og dato, ……..............................

----------------------------------------

Underskrift
