# Supplementary material for: Cardiac function in newborns of obese women and the effect of exercise during pregnancy. A randomized controlled trial
Source: PLoS One. 2018 Jun 1;13(6):e0197334. doi: 10.1371/journal.pone.0197334 (PMC5983429; doi:10.1371/journal.pone.0197334)
Supplement: S5 Text — (PDF) [file pone.0197334.s008.pdf]

NTNU
Det skapende universitet

HELSE MIDT-NORGE

Forespørsel om å delta som **kontroll** i forbindelse med en vitenskapelig undersøkelse ”Trening under svangerskapet”

Studien er et samarbeidsprosjekt mellom NTNU og Kvinneklinikken ved St. Olavs Hospital

Bakgrunn og målsetting
I forbindelse med et forskningsprosjekt som pågår ved St. Olavs Hospital hvor vi studerer overvektige gravide, ønsker vi å sammenlikne med friske normalvektige kvinner.

Hvem kan delta, og hva innebærer deltakelse
Gravide kvinner (≥18 år) med kroppsmasseindeks* ≤ 25 inviteres til å delta. Vi ønsker å vite mer om den normale utviklingen i svangerskapet når det gjelder hjertefunksjon hos fostret og mor. Testingen foregår ved St. Olavs Hospital. Vi vil se på hjertet (både i hvile og ved anstrengelse) og blodårene dine med ultralyd og registrere vekt. Barnets hjertefunksjon vil bli undersøkt litt nøyere enn ved vanlig oppfølging, ved ultralyd i uke 14, 20 og 32. Mors hjertefunksjon blir undersøkt samtidlig. I tillegg vil barnet bli hjerteundersøkt mens mor og barn er innlagt på barselavdelingen, samt 6-8 uker etter fødsel. Ultralyd av mors blodåre blir gjort i uke 14 og 36.
Dessuten ber vi om at vi får benytte informasjon om vekt og blodtrykk fra svangerskapsjournalen, og opplysninger om fødselsforløpet og barnets vekt, lengde samt rutine barnelegeundersøkelse fra fødselsjournalen.

Kroppsmasseindeks beregnes ved:

$$\frac{\text{Vekt (kg)}}{\text{Høyde (m) x høyde (m)}}$$

Eksempel: 84kg/1,65 m x 1,65 m = 31

NTNU
Det skapende universitet

HELSE MIDT-NORGE

Frivillighet og samtykke

- Deltakelse i prosjektet er frivillig.
- Alle deltakere i prosjektet har rett til å trekke seg fra prosjektet når de måtte ønske, uten at dette får konsekvenser for videre oppfølging og behandling. All informasjon deltakerne gir i forbindelse med prosjektet, behandles konfidensielt, og data avidentifiseres. Alle som skal ha kontakt med de innsamlede data, er underlagt taushetsplikt i henhold til Forvaltningslovens § 13 og Helsepersonellovens § 21.
- Deltakerne er dekket av Pasientskadeerstatningsordningen.

Etisk og faglig vurdering

- Prosjektet er vurdert av Regional komité for medisinsk forskningsetikk, Region Midt-Norge, og komiteen har godkjent at prosjektet gjennomføres.

Ansvarlige prosjektledere er Charlotte Björk Ingul, lege og forsker på NTNU.

HVIS DU ØNSKER Å DELTA, ELLER HAR SPØRSMÅL OM PROSJEKTET, BES DU KONTAKTE:

Charlotte Björk Ingul
Du kan ta kontakt på følgende måte:

- E-post: charlotte.b.ingul@ntnu.no
- Telefon: 95805886

NTNU
Det skapende universitet

HELSE MIDT-NORGE

ID-nummer:

(fylles ut av prosjektkoordinator)

Hvis du ønsker å delta må du fylle ut dette samtykkeformularet. Samtykkeformularet leveres til prosjektkoordinator ved oppmøte for første test.

SAMTYKKEERKLÆRING om å delta som KONTROLL i forbindelse med en vitenskapelig undersøkelse ”Trening under svangerskapet”

Jeg har lest informasjonsskrivet og har hatt anledning til å stille spørsmål. Jeg er også informert om at journalopplysninger fra det aktuelle svangerskap og fødsel vil bli gjennomgått og registrert og samtykker i å delta i studien.

Sted og dato, ……...............................

Underskrift
